# Competitive Dynamics in MSTd: A Mechanism for Robust Heading Perception Based on Optic Flow

**DOI:** 10.1371/journal.pcbi.1004942

**Published:** 2016-06-24

**Authors:** Oliver W. Layton, Brett R. Fajen

**Affiliations:** Department of Cognitive Science, Rensselaer Polytechnic Institute, Troy, New York, United States of America; Technische Universitat Chemnitz, GERMANY

## Abstract

Human heading perception based on optic flow is not only accurate, it is also remarkably robust and stable. These qualities are especially apparent when observers move through environments containing other moving objects, which introduce optic flow that is inconsistent with observer self-motion and therefore uninformative about heading direction. Moving objects may also occupy large portions of the visual field and occlude regions of the background optic flow that are most informative about heading perception. The fact that heading perception is biased by no more than a few degrees under such conditions attests to the robustness of the visual system and warrants further investigation. The aim of the present study was to investigate whether recurrent, competitive dynamics among MSTd neurons that serve to reduce uncertainty about heading over time offer a plausible mechanism for capturing the robustness of human heading perception. Simulations of existing heading models that do not contain competitive dynamics yield heading estimates that are far more erratic and unstable than human judgments. We present a dynamical model of primate visual areas V1, MT, and MSTd based on that of Layton, Mingolla, and Browning that is similar to the other models, except that the model includes recurrent interactions among model MSTd neurons. Competitive dynamics stabilize the model’s heading estimate over time, even when a moving object crosses the future path. Soft winner-take-all dynamics enhance units that code a heading direction consistent with the time history and suppress responses to transient changes to the optic flow field. Our findings support recurrent competitive temporal dynamics as a crucial mechanism underlying the robustness and stability of perception of heading.

## Author Summary

Humans have little difficulty moving around in dynamic environments containing other moving objects. Previous research has demonstrated that moving objects may induce biases in perceived heading in some circumstances. Nevertheless, heading perception is surprisingly robust and stable. Even when large moving objects occupy much of the visual field and block our view of the future path, errors in heading judgments are surprisingly small—usually less than several degrees of visual angle. Furthermore, perceived heading does not abruptly shift or fluctuate as moving objects sweep across the observer’s future path. The aim of the present study is to investigate the qualities of our visual system that lead to such robust heading perception. We simulated two existing models that specify different heading mechanisms within the visual system and found that they could not capture the robustness and stability of human heading perception in dynamic environments. We then introduced the *competitive dynamics model* that succeeds due to its reliance on recurrent, competitive interactions among neurons that unfold over time that stabilize heading estimates. Our results suggest that competitive interactions within the visual system underlie the robustness and stability of human heading perception.

## Introduction

Humans move through an often cluttered world with ease. We effortlessly walk and drive through busy streets in everyday life without colliding with other moving pedestrians. These competencies require an accurate, reliable, and stable perception of the direction of self-motion (i.e., heading). Although heading perception is inherently multisensory, with contributions from the vestibular [1–3] and motor [4] systems, vision represents the dominant sensory modality for many animals [5–7]. Forward self-motion along a linear trajectory produces a field of radially expanding optic flow that emanates from a singularity known as the focus of expansion (FoE), which coincides with the direction of travel in the absence of eye movements.

It is well established that the primate visual system is sensitive to and uses information in optic flow to perceive heading. Single neurons in the dorsal medial superior temporal area (MSTd) [8–10], ventral intraparietal area (VIP) [11,12], and other brain areas, are tuned to the direction of self-motion through three-dimensional (3D) space. Such neurons are sensitive to radial fields of motion with different FoE positions that encompass much or all of the visual field that is experienced during self-motion [13,14]. At the population level, the largest proportion of neurons is tuned to FoE positions that correspond to straight-ahead headings [10], which is to be expected for a system that depends on optic flow to perceive the direction of self-motion during locomotion.

Much of what is known about heading perception comes from psychophysical experiments wherein human subjects view computer displays of simulated self-motion and judge the perceived direction of travel once the trial concludes. Remarkably, humans can judge their heading to within 1–2°, even when the environment contains as few as ten dots [15,16]. Heading judgments remain accurate despite the presence of substantial amounts of noise or the removal of large portions of the flow field [17,18]. The accuracy of human heading judgments is consistent with the tuning of MSTd neurons that shows maximal sensitivity around the straight ahead [2,19].

### The robustness and stability of heading perception

**Fig 1:**
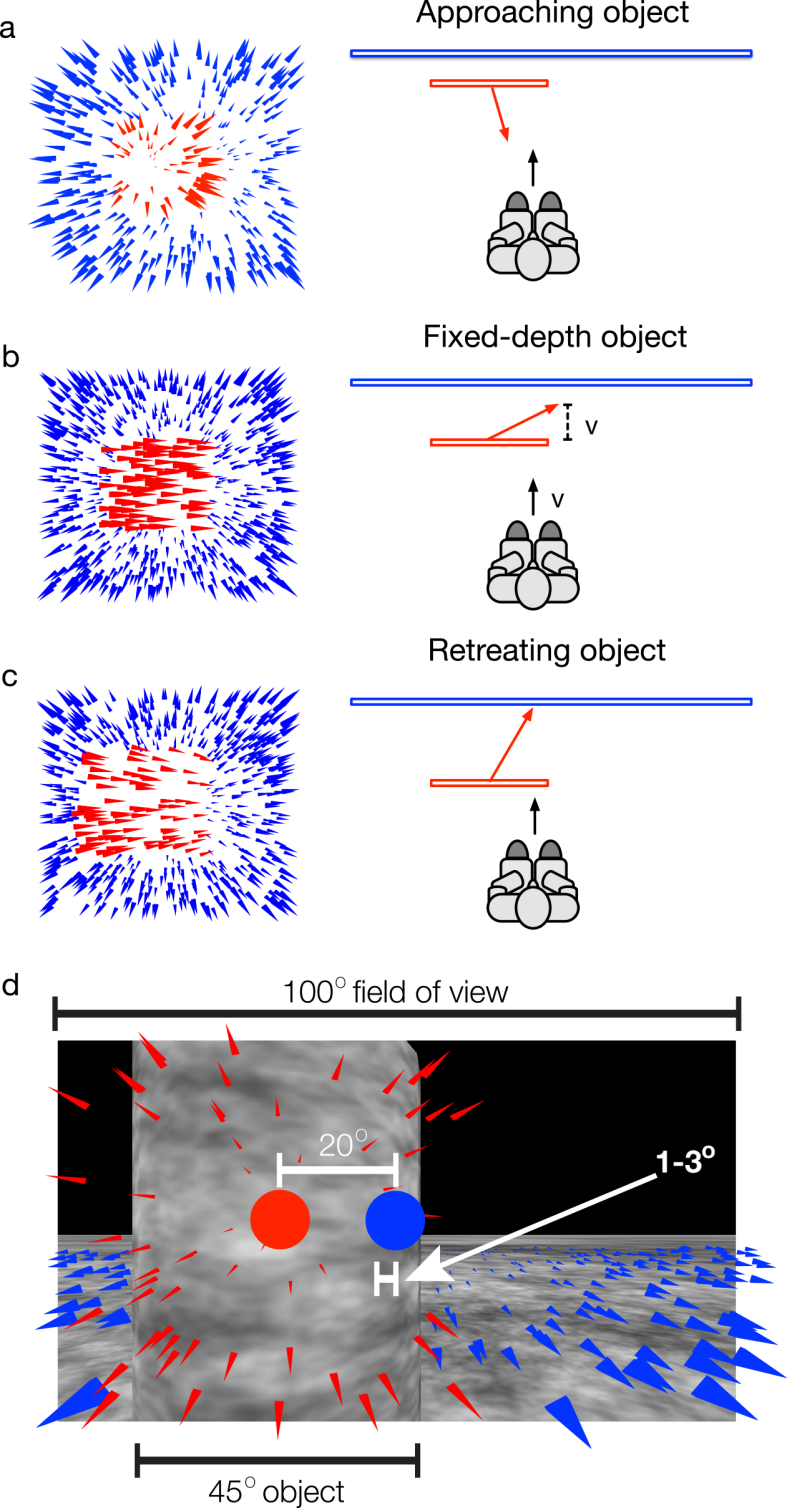
**Overview of the cases in which an object may cross the future path of a moving observer (right panels) and corresponding optic flow fields (left panels).** In panels on the left, red and blue arrowheads correspond to optic flow from the object and background, respectively. In the right panels, the black arrow shows the heading direction of the observer and red arrows show the movement direction of the object. In panel b, the fixed-depth object moves in depth at the same rate as the observer (*v*). (d) A depiction of self-motion in the presence of an approaching moving object that occupies much of the visual field (45° of the 100° field of view). Even though the FoE due to the observer self-motion relative to the background (blue disk) and to the object (red disk) are separated by 20°, the heading bias is quite small—only 1–3°.

Heading perception is not only accurate, it is also remarkably robust and stable. These qualities warrant further investigation and are the focus of the present study. The robustness and stability of heading perception are especially evident in dynamic environments containing independently moving objects. Regions of the optic array corresponding to moving objects generally contain optic flow that is inconsistent with the background optic flow and uninformative about heading. Nonetheless, heading perception is biased by moving objects by no more than a few degrees. Objects that approach the observer in depth (*approaching objects*; Fig 1A) induce a bias in the direction opposite the object motion of ~3° [20]. When objects maintain a fixed depth with respect to the observer as they move laterally (*fixed-depth objects*; Fig 1B), heading perception is biased by ~1° in the direction of object motion [21]. Objects that recede in depth from the observer as they move across the observer’s future path (*retreating objects*; Fig 1C) yield a heading bias in the direction of object motion of less than 3° (Layton & Fajen, in preparation).

These biases are surprisingly small when one considers the conditions in which they are induced. The moving objects in the aforementioned experiments were generally large and moved near the observer, such that a sizeable proportion of the visual field contained discrepant optical motion. They often crossed the observer’s future path, thereby occluding the region of the optic array near the background FoE (as in Fig 1), which is known to be the most informative region for heading perception [16,22]. Moving objects that approach the observer in depth generate a radial pattern of optic flow with a FoE of their own that may be offset from the background FoE by much more than a few degrees. In some circumstances, all of these potential complications may occur at the same time. In Fig 1D, for example, the moving object occupies approximately half of the visual field, occludes the background FoE (indicated by the blue dot), and generates radial motion with a FoE (red dot) that is offset from the background FoE by 20°. The fact that heading perception under these conditions is biased by no more than a few degrees attests to the robustness of the visual system.

Furthermore, such biases are induced only when objects cross or move near the observer’s future path [20,23]. Objects that move far away from the future path do not influence heading perception. Nonetheless, our experience when an object approaches and eventually crosses our future path is not that heading abruptly shifts; that is, humans do not perceive themselves as moving in one direction at one instant and then in a different direction at the next instant when the object begins to cross the path. Heading perception is more stable and less susceptible to fluctuations.

Previous research on heading perception in the presence of moving objects [20,21,24] has focused on the sources of bias. The fact that heading perception is as reliable and stable as it is under such conditions has been largely overlooked. Nonetheless, these qualities of heading perception are worthy of investigation. Understanding the mechanisms that underlie the robustness and stability of heading perception was the primary aim of the present study.

### Temporal dynamics and robust heading perception

We test the hypothesis that the robustness and stability of heading perception is rooted in a particular form of temporal dynamics within the visual system—specifically, recurrent competitive interactions that unfold over time among units in area MSTd. As a simple demonstration that heading perception has an important temporal component, consider a scenario in which a moving object approaches and crosses the observer’s future path from the left or right. Recall that heading perception is biased under these conditions but that the bias is surprisingly small. One possible reason why the bias is not larger than it is, is that heading perception is based on the temporal evolution of the optic flow field, including not only the period of time when the object crossed the path but also prior to this point, before the more informative regions of the flow field were occluded by the object. Although this may seem obvious, existing models of heading perception [20,25] have no features for capturing heading perception as a process that evolves over time. As we explain below, these models estimate heading based on the instantaneous flow field and generate a new estimate that is independent of the previous one at each successive instant.

We tested the role of temporal dynamics in an experiment in which human subjects made heading judgments in the presence of an object that approached from the side and crossed the observer’s path at the end of the trial [23]. Stimulus duration was varied between 150 and 1500 ms. Importantly, within each object trajectory condition, the last 150 ms was the same across stimulus durations, but conditions with longer durations also included the earlier part of the event leading up to the last 150 ms prior to occlusion of the background FoE. If heading perception is based on the temporal evolution of the optic flow field, the heading bias should be weaker in the longer duration conditions because the visual system should be able to use the information from the earlier part of the trial—before the object occluded the path—to improve the accuracy of the estimate.

Indeed, when stimulus duration was short (i.e., when subjects only saw the last part of the trial), they exhibited a very large heading bias (~6°). However, the bias was dramatically reduced when stimulus duration was longer–that is, when the earlier part of the trial before the object crossed the path was also included in the stimulus. The findings indicate that heading perception is based on the evolution of the optic flow field and the ability to integrate information over time underlies the surprising accuracy and stability of heading perception. In other words, temporal dynamics offers a candidate solution to the problem posed above about why heading perception is not more biased by moving objects than it is, and why we do not experience abrupt transitions in perceived heading.

### Aim and approach of the present study

These findings provide compelling evidence that heading perception is based on the evolution of the optic flow field, but are not especially informative about the nature of the underlying neural mechanisms. In the present study, we used modeling and simulation to test the sufficiency of a particular type of mechanism involving on-center/off-surround recurrent interactions among MSTd neurons. Neurons in a recurrent network send inhibitory feedback to other neurons to balance the excitatory feedback they send themselves to potentiate their activity. Recurrent dynamics among neurons in MSTd are compatible with the neuronal decay time constant in MSTd (81 msec), which is as much as five times slower than that of areas from which MSTd receives input and indicates a persistence in the heading signal long after the visual optic flow signal ceases [26]. Competition among heading-sensitive neurons in MSTd over time through recurrent interactions may exert nonlinear effects on the heading signal—the contrast of the heading signal could be enhanced and the uncertainty about heading reduced. In the language of Bayesian inference, MSTd neurons may update the network’s belief about heading over time [27]. As we demonstrate below, these interactions that unfold over time may serve as a mechanism to stabilize heading perception, even when the visual signal is temporarily disrupted.

Our approach is to compare the performance of a model with recurrent temporal dynamics in MSTd against two models without this property, focusing on whether these models capture the spatio-temporal robustness of human heading perception in dynamic environments. The model with recurrent temporal dynamics is an updated version of the model introduced by Layton, Browning, & Mingolla [28]. The other two models are the *motion pooling* model developed by Warren & Saunders [20] and the *differential motion* model developed by Royden [25]. These models were chosen because they are representative of existing biological modeling approaches and because they were designed to estimate heading in the presence of independently moving objects. In all three models, the similarity is computed between the optic flow field (or a transformation thereof) and a number of vector field templates containing radial expansion with different FoE positions. Each template resembles the canonical receptive field organization of a MSTd cell selective to a particular FoE location in the visual field and is center-weighted—motion vectors nearby the preferred FoE position are weighted greater than those located further away. Such templates have characteristics that are consistent with those that develop in models using supervised and unsupervised learning [29–32]. Motion pooling models have demonstrated that matching these biologically inspired global motion templates with the patterns of optic flow that arise during self-motion provides a plausible means for cells in MSTd to extract heading [33–36] (but see [37]). The template match feeds MSTd units with their inputs and the preferred FoE position of the most active unit reflects the heading estimate of the model.

The differential motion model [25] is distinct from the others in that the template match is performed on a field of difference vectors rather than the optic flow field. Differential motion models were originally proposed to account for the ability to perceive heading while making eye movements, which introduce rotation into the flow field. Any instantaneous optic flow field can be decomposed into translational and rotational components [38]. A vector’s translational component depends on the corresponding point’s depth in the environment, whereas the rotational component does not. Therefore, subtracting nearby motion vectors that correspond to points at different depths within the environment eliminates the rotational component and results in a scaled version of the translational component. Because the translational component is informative about the observer’s heading and the rotational component is not, certain difference vectors may be used to recover a heading estimate. Rieger & Lawton [39] developed the first differential motion algorithm to compute heading based on a decomposition of translational and rotational flow when differential motion parallax is present. Hildreth [40] later extended the approach with a voting procedure to account for the presence of moving objects. A number of neural models that decompose flow into translational and rotational components have successfully simulated properties of MT and MST [41,42] (but see [43]).

The differential motion model developed by Royden and simulated in the present study [25] is a refinement of an earlier version [44] to situate the differential motion algorithm in a biological framework. The field of difference vectors in the Royden model is obtained by processing the optic flow field with motion sensitive units with antagonistic surrounds whose properties resemble those of cells in primate MT^—^. These operators respond optimally when a sharp change in speed occurs within the receptive field along the preferred motion direction, which may coincide with a sudden change in depth and result in motion parallax: near background motion results in a faster optical speeds than far background motion. The heading estimate in the differential motion model is the direction that corresponds to the preferred FoE of the most active center-weighted template.

Both the motion pooling model [20] and the Layton et al. model (as well as its successor introduced here) compute the template match directly on the optic flow field. We refer to the latter as the *competitive dynamics* model to highlight its unique feature—that it is a dynamical system that continuously integrates optic flow within a competitive network of MSTd neurons [28]. This differs from the differential motion and motion pooling models, which only process vector fields at independent points of time.

Two other models, neither of which can be classified as motion pooling or differential motion models, warrant mentioning. First, the analytic model of Raudies & Neumann [45] relies on neither motion differences nor templates to account for the pattern of human heading biases in the presence of moving objects, but rather a weighted combination of segmentation cues derived from the flow field. Heading bias arises in the model even without segmentation cues because the moving object induces a discrepancy compared to analytic parameters that describe the observer’s self-motion in a static environment. The pattern of bias produced by the model does not resemble that of humans, but segmenting the optic flow field by accretion/deletion, expansion/contraction, and acceleration/deceleration improves the correspondence. Second, Saunders & Niehorster [46] cast the problem of estimating heading in the presence of moving objects into a Bayesian context whereby the objective is to estimate the translational and rotational components of an ideal observer from optic flow along with the depth of points in the scene. The model estimates the posterior probability that an observer moves along a particular heading by multiplying the likelihoods that each motion vector in the optic flow pattern was independently generated by a particular combination of observer translation and rotation parameters. The model accounts for human heading bias in the presence of approaching and fixed-distance objects. We will not give further consideration to either model in our simulations below because both process vector fields at independent points of time and because our focus in the present study is on neural models.

We simulated the differential motion, spatial pooling, and competitive dynamics models under a variety of conditions to test for robustness and stability in heading estimates (see Methods section for details about the models and simulations). To anticipate the results, we found that the differential motion and spatial pooling models yield erratic, sometimes wildly fluctuating heading estimates over time. Furthermore, simply adding temporal smoothing of optic flow signals to these models does not capture the spatio-temporal characteristics of human heading perception. In contrast, the estimates from the competitive dynamics model are less biased by moving objects, less variable, more stable, and more similar to human heading estimates in the presence of moving objects. Taken together, the findings imply that competitive interactions within MSTd are a plausible mechanism to account for the robustness and stability of human heading perception.

## Results

### Differential motion and motion pooling model simulations

Previous efforts to evaluate the differential motion and motion pooling models have focused on how accurately they reproduce patterns of human heading judgments [20,25,28]. Both models succeed in capturing the heading bias in humans for approaching objects, but only the differential motion model has been shown to match human judgments for fixed-depth objects. It has been argued that the motion pooling model fails to capture the human heading bias for fixed-depth objects [25,28], but this has not actually been formally tested. Neither model has been evaluated in the retreating object scenario. More importantly, the robustness and stability of these models has never been systematically explored.

### Approaching, fixed-depth, and retreating objects

**Fig 2:**
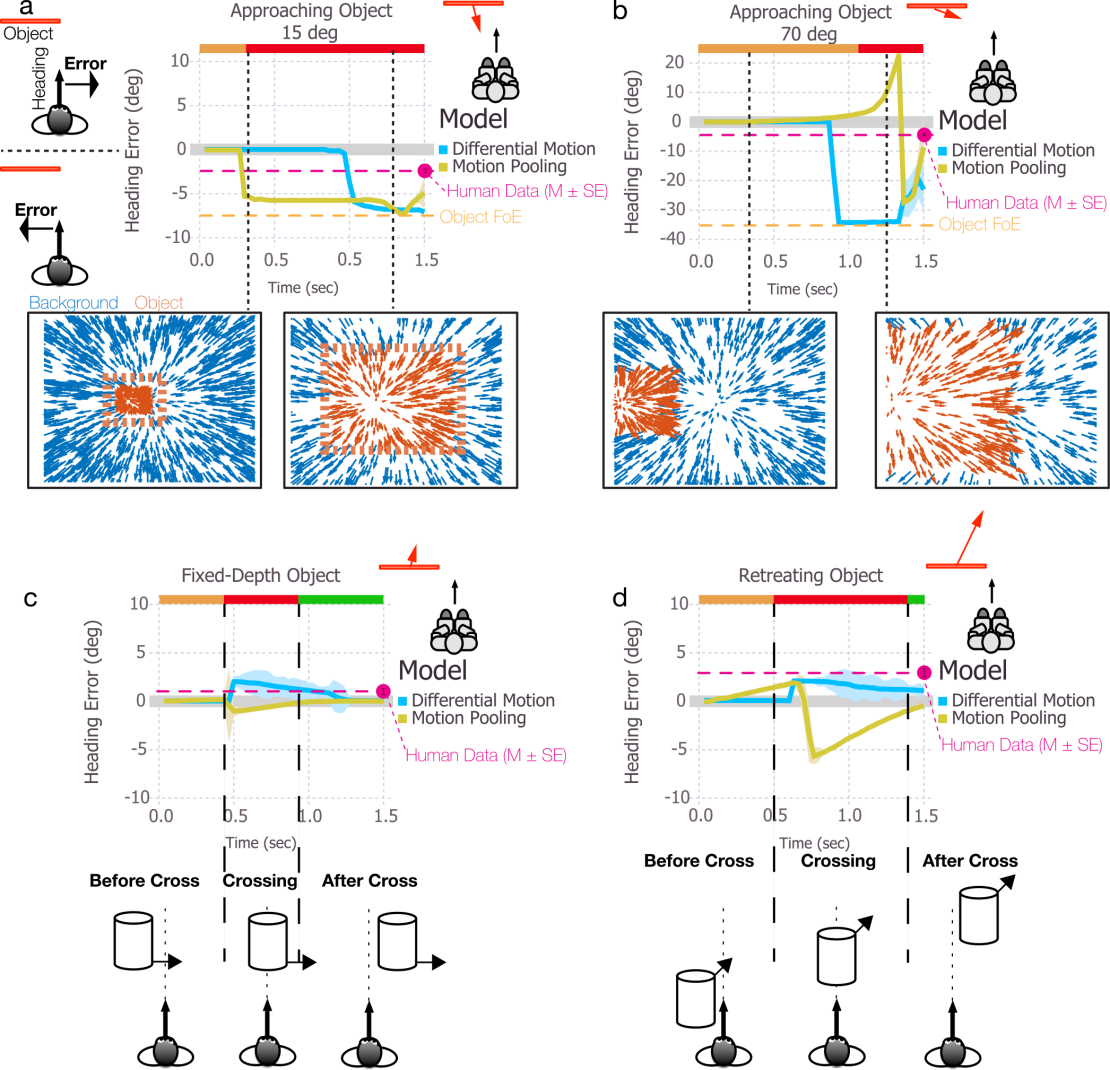
Simulations of the differential motion and motion pooling models of self-motion in the presence of an object that moves independently of the observer over 1.5 sec. In (a-b), the object approaches the observer at 15° and 70°, respectively. In panel c, the object moves to the right while maintaining a fixed depth relative to the observer, and in panel d, the object retreats at a 56° angle. Heading estimates produced by the differential motion and motion pooling models are indicated by blue and gold, respectively. Colored bands surrounding each curve indicate ±1 standard error of the mean (SEM). In all conditions, the object started to the left of the observer’s heading. Error is defined as negative and positive for estimates that deviated from the heading direction in the direction opposite object motion or in the same direction as object motion, respectively. The orange, red, and green bars at the top of each subplot indicate the periods of time before, while, and after the object crosses the observer’s future path. Magenta horizontal dashed lines indicate mean human heading judgments from psychophysical data in similar circumstances, where heading is judged at the end of the trial (1.5 sec; error bars indicate ±1 SEM). The positions of the object FoE (orange dashed line) are 7.5° (a) and 35° (b).

In this section, we examine model estimates of heading during self-motion in the presence of moving objects that cross the observer’s future path while approaching (Fig 2A and 2B), maintaining a fixed-depth (Fig 2C), or retreating (Fig 2D). The blue and gold curves in each plot show the mean heading error over time for the differential motion and motion pooling models, respectively, with lighter shaded regions indicating ±1 SE. The color of the horizontal bar at the top of each subplot in Fig 2 indicates when the moving object is crossing the observer’s future path (red), as well as the portions of the trial before (orange) and after (green) crossing.

First, we consider simulations with an approaching object that crosses the observer’s future path at a 15° angle (7.5° object FoE offset) (Fig 2A). The typical bias in human judgments in the presence of objects that approach at comparable angles is about 2.5° in the direction opposite object motion [23]. Note that subjects in the human experiments made judgments after viewing the entire stimulus, so the existing data are not informative about how perceived heading evolves over time as the object changes position in the visual field. As such, we represent typical human performance in Fig 2 using a single dot positioned at the far right of the figure with a dashed line of the same color for reference.

The motion pooling model initially yields unbiased heading estimates but quickly exhibits a ~6° bias in the direction opposite of object motion as the object approaches and crosses the future path. This is consistent with the human data in direction but greater in magnitude. The heading bias remains relatively stable while the object occludes the background FoE. The differential motion model also yields a bias (~7°) in the direction opposite object motion during object crossing, but the bias arises later. That is, the differential motion model yields accurate heading estimates for a longer period of time while the object occludes the heading direction compared to the motion pooling model.

Although the heading biases from both models are only slightly greater than those exhibited by humans, model performance deviates from human judgments much more dramatically at larger object trajectory angles. When the object approaches along a 70° angle (35° object FoE offset), the differential motion model exhibits a bias that exceeds that of humans by a factor of 10 (Fig 2B). The motion pooling model yields a large bias in the direction of object motion, followed by a dramatic reversal in the opposite direction. The large initial bias in the direction of object motion (positive in Fig 2B) was unexpected because the motion pooling model is known to exhibit heading bias in the opposite direction for approaching objects (i.e., toward the object FoE or negative in Fig 2A) [20,24]. The positive bias arises from the strong rightward radial motion of the background flow ahead of the leading edge of the moving object, which activates templates weighted to the far right of actual heading. The level of activation is only moderate because the rightward flow is not a perfect match for templates in that direction. Nonetheless, the activation level is higher than for other templates, including those more closely aligned with the object FoE. This is because as the object draws closer in depth, the spatial distribution of dots nearby the object FoE becomes sparser. Radial templates weighted nearby the object FoE are only weakly activated because of the limited amount of motion. Eventually, as the object continues to cross the observer’s path, it occludes enough of the background flow to diminish activation of templates weighted to the far right. At this point, the most active templates are those closely aligned with the object FoE, causing the heading estimate to abruptly reverse and exhibit a bias in the direction opposite object motion.

Although the direction of the bias generated by the motion pooling model was initially unexpected, the magnitude of bias in both models is not surprising given that both models estimate heading based on the instantaneous flow field, which is dominated by discrepant object motion toward the end of the trial. Nonetheless, human heading judgments are far more robust even when objects approach at larger angles [24].

Fig 2C shows the simulation results with a fixed-depth object. Both the differential motion model and motion pooling model yield unbiased or weakly biased heading estimate before and after the objects crosses the observer’s path. During the crossing period, the differential motion model produces a 1–2° heading bias in the direction of object motion, consistent with human heading judgments. On the other hand, the motion pooling model produces a weak bias in the direction opposite object motion, which is not consistent with human heading judgments. Variability is slightly larger in the differential motion model, except for the spike that occurs in the motion pooling model estimates when the object begins to cross the future path.

Fig 2D depicts the simulation results for the retreating object scenario. The differential motion model yields accurate heading estimates until shortly before the object crosses the path, at which point there is a sharp rise in the bias. The 1–2° bias in the direction of object motion is consistent with the human data (Layton & Fajen, in preparation). Model variability is comparable to that obtained for the fixed-depth object. The heading error generated by the motion pooling model gradually ramps up while the object is approaching the observer’s path, and then sharply reverses to a bias in the direction opposite object motion. The reversal and subsequent gradual bias reduction occur because templates in the model respond to the radial-like motion pattern created by the trailing edge of the object and the background. The most active template in the model tracks the position of trailing edge, which progressively moves toward the heading direction, resulting in a weakening of the bias.

### Stability of model estimates and human heading perception

Although the mean estimates from the differential and motion pooling models match those of human observers in some conditions, the model and human estimates differ dramatically in direction and/or magnitude under other conditions. Furthermore, these models do not exhibit the stability that is characteristic of human heading perception. Heading estimates from both models often changed very abruptly, increasing or decreasing by many degrees of visual angle in less than 100 ms. If human heading perception was subject to such wild fluctuations, moving objects would induce easily noticeably shifts in perceived heading as they approach and cross the future path. Yet both psychophysical studies and introspection while driving or walking in busy environments suggest that heading perception is far more stable and that any changes in perceived heading are small and too gradual to be noticed.

### Temporal smoothing

**Fig 3:**
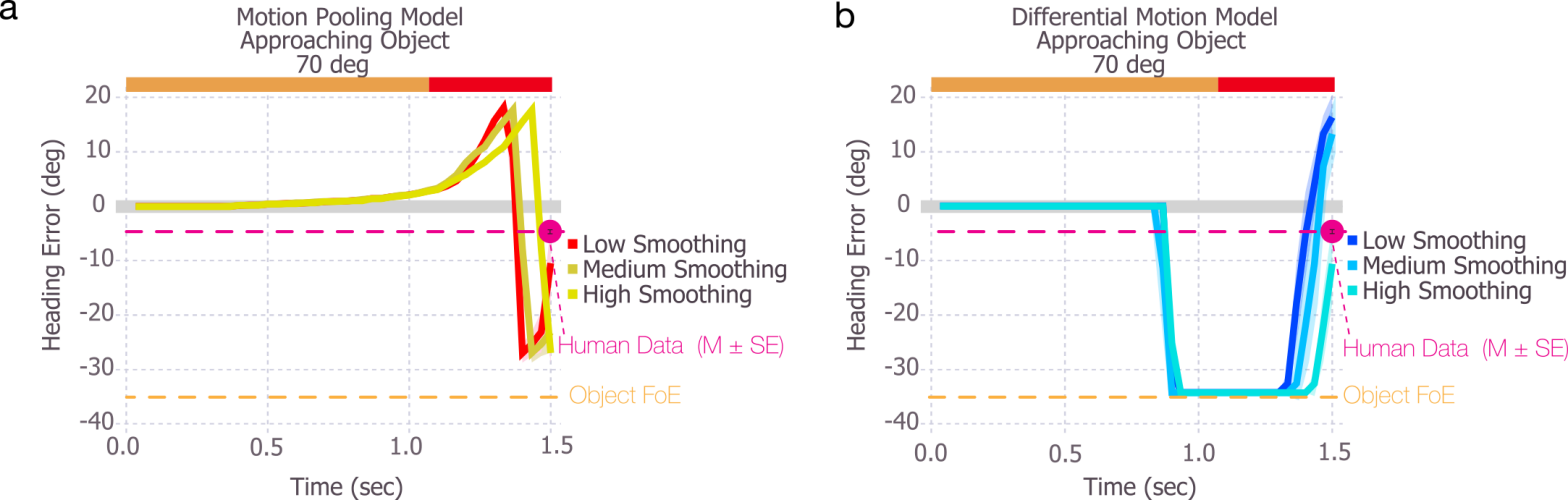
The addition of temporal smoothing does not improve the abrupt changes in heading estimates produced by the differential motion and motion pooling models. The models simulated the 70° angle of approach condition (Fig 2B) with low, medium, and high amounts of temporal averaging of activation in model MST.

Because the differential motion and motion pooling models were not designed to integrate optic flow over time, we explored whether smoothing the activation of model MSTd units over time with a moving average would address some of the issues with the stability of heading estimates. This was implemented by applying a 3 (‘Low’), 6 (‘Med’), or 9 (‘Hi’) frame moving average to the activation produced by each unit in the 2D MSTd array. As illustrated in Fig 3, temporal smoothing was not effective. In both models, the large heading biases, abrupt changes, and reversals remained even with a high degree of temporal smoothing. Of course, the degree of smoothing could be further increased, but that would not qualitatively change the estimates and would introduce significant lag into the signal, making the model sluggish in response to actual changes in heading.

### The competitive dynamics model

We now introduce the competitive dynamics model, which is based on the model of Layton et al. [28], and explore whether it better captures the robustness and stability of human heading perception. The model contains areas that correspond to the primate retina, lateral geniculate nucleus (LGN), primary visual cortex (V1), medial temporal area (MT^+^), and the dorsal medial superior temporal area (MSTd) (see Methods for details). These areas are organized into three functionally distinct stages: sensitivity to change in the retina; motion detection in LGN, V1, and MT^+^; and self-motion estimation in MSTd. The self-motion estimation mechanisms are the same as those in the model of Layton et al. [28], but the stages for sensitivity to change and motion detection are new. As a large dynamical system, populations of neural units in each area obey systems of Hodgkin-Huxley-like ordinary differential equations. In other words, model cells in each area temporally integrate the network response to the optic flow time history and the bottom-up signal derived from the presently available optic flow.

The main feature that differentiates the competitive dynamics model from the differential motion and motion pooling models is the use of recurrent competitive dynamics among model MSTd cells that unfold over time. Units in model MSTd obey on-center/off-surround recurrent competitive dynamics, which refine the heading estimate over time. Each unit competes for its heading representation: a unit enhances the signal for its preferred heading through self-excitation and suppresses other heading signals generated through the pattern of activity produced by other units in the network. As a dynamical system, the network takes time to develop a reliable heading estimate, which persists for some time—even if the optic flow signal is interrupted. The competitive dynamics that refine the heading estimate and the persistence of activity over time in the competitive dynamics model may hold the key to robust heading perception.

### Approaching, fixed-depth, and retreating objects

**Fig 4:**
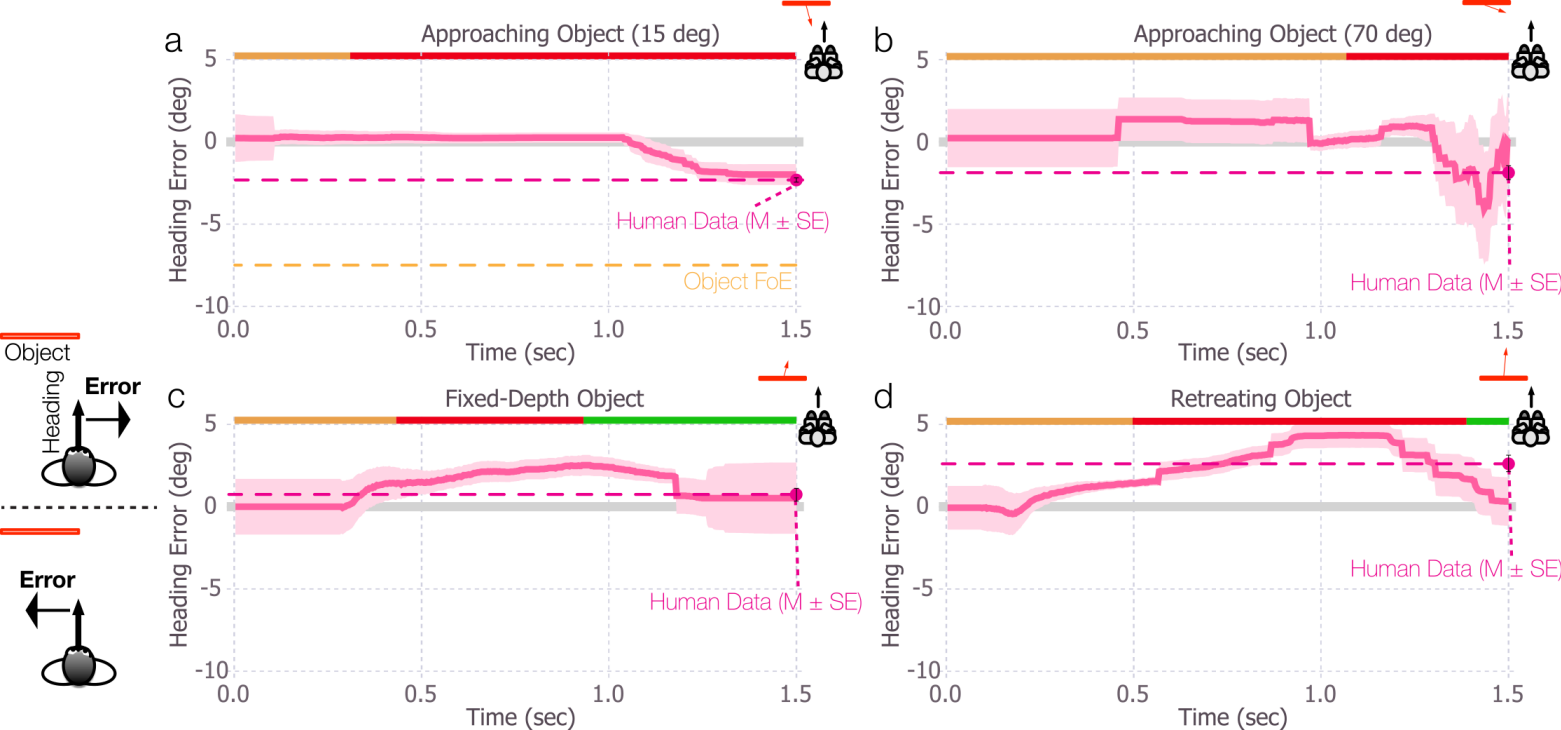
Simulations of the competitive dynamics model in conditions that correspond to those in Fig 2. Colored bands surrounding each curve indicate ±1 standard error of the mean (SEM). In all conditions, the object started to the left of the observer’s heading. Error is defined as negative and positive for estimates that deviated from the heading direction in the direction opposite object motion or in the same direction as object motion, respectively. Error bars for the mean human heading judgments indicate ±1 SEM.

To test the performance of the model, we ran simulations under the same conditions used to test the previous models. In Fig 4, we plot the mean heading error produced by the competitive dynamics model for the approaching, fixed-depth, and retreating object conditions. The mean heading bias reaches ~2.5° in the direction opposite object motion for the object approaching along a 15° angle, ~4° in the same direction for an object approaching along a 70° angle, ~2.5° in the direction of object motion for the fixed-depth object, and ~4° in the direction of object motion for the retreating object. Like the differential motion model, the competitive dynamics model yields heading biases that consistently match those of human observers in direction. Unlike the differential motion and motion pooling models, however, heading error builds up in each condition over several hundred milliseconds and does not exhibit large, sudden excursions or reversals in the direction of bias. Furthermore, the competitive dynamics model yields far more accurate, stable, and human-like heading estimates when objects approach at extreme angles (e.g., 70°) compared to the differential motion and motion pooling models (compare Figs 4B and 2B, noting the difference in the scale of the y-axis). In summary, the competitive dynamics model better replicates the complex pattern of human heading biases across variations in object trajectories, and yields heading estimates that are relatively stable and change gradually.

**Fig 5:**
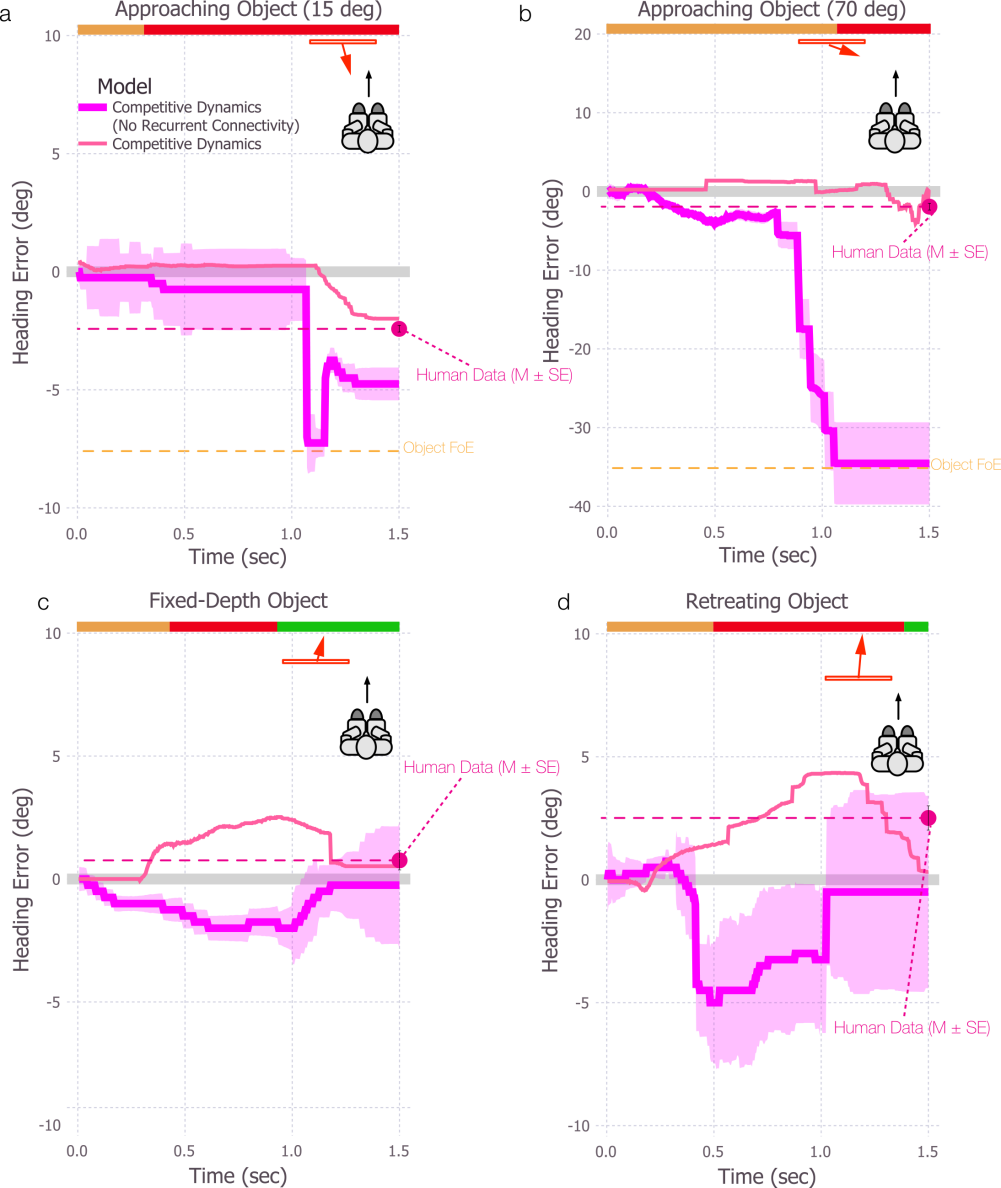
Simulations of the competitive dynamics model with (thin pink curve) and without (thick fuscia curve) recurrent connections in model MSTd in conditions that correspond to those in Fig 2. The modified version of the model lacked any term in Eq 21 that involves *f*(∙) or *g*(∙). Colored bands surrounding the thick pink curve indicate ±1 standard error of the mean (SEM); bands around the thin pink curves appear in Fig 4 and are omitted here for visual clarify. Error bars for the mean human heading judgments at the end of the trial also indicate ±1 SEM. In all conditions, the object started to the left of the observer’s heading. Error is defined as negative and positive for estimates that deviated from the heading direction in the direction opposite object motion or in the same direction as object motion, respectively. Compared to the regular model, lesioning recurrent connections in model MSTd introduced instability into heading estimates overall and in the presence of both approaching objects, heading estimates were strongly influenced by the object FoE. The lesioned model could not account for the direction of the human heading bias in the fixed-depth and retreating object scenarios. Weak matches to expansion on the interior of the retreating object and the lack of interaction between expansion and contraction cells raised heading estimate variability.

Before we can conclude that the improvement in performance is due to recurrent competition, it is necessary to rule out other differences between the competitive dynamics model and the differential motion and motion pooling models. As such, we simulated a version of the competitive dynamics model without connectivity between model MSTd units. The thick fuchsia curves in Fig 5 depict the model performance on the approaching, fixed-depth, and retreating objects simulations when we lesioned connections within model MSTd. Without recurrent competition, heading estimates change more abruptly, are strongly influenced by the object FoE in the presence of the objects that approach along 15° and 70° trajectories, and are in the incorrect direction in the presence of the fixed-depth and retreating objects. Together, these findings support the hypothesis that competitive interactions in MSTd play a crucial role in the robustness of human heading perception.

### Additional tests of robustness and stability

#### The pseudo FoE effect

**Fig 6:**
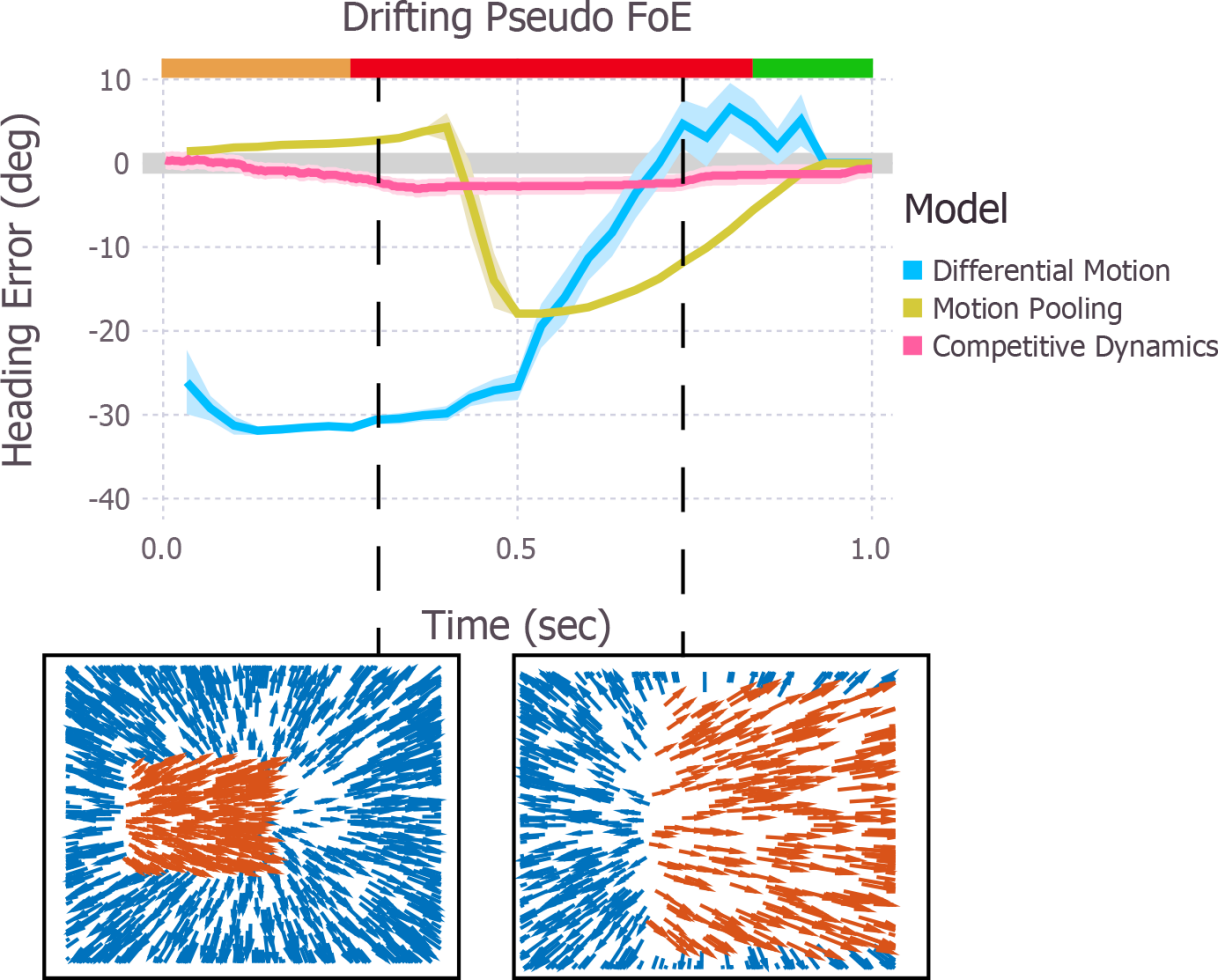
Simulations of an approaching object that creates a radial-like flow pattern nearby the object’s trailing edge (pseudo FoE). The pseudo FoE sweeps from left to right on the left side of the visual field until it approximately coincides with the heading direction (center of the screen) at the end of the trial. The pseudo FoE dominates heading estimates in the motion pooling and differential motion models, which yield large, rapidly changing heading biases. The competitive dynamics model collects evidence about heading over time and the transient rapid sweeping of the pseudo FoE only weakly affects the heading estimate.

Temporal dynamics could play an important role in the robustness and stability of heading perception in other scenarios involving moving objects. The insets at the bottom of Fig 6 depict the optic flow field generated when an approaching object crosses the observer’s path from left to right. Notice that the optical motion near the trailing edge of the object is radial-like—the motion is rightward within the object and leftward behind the trailing edge, and upward and downward above and below the horizon, respectively. Although the motion to the left and right of the trailing edge radiates from different locations (rather than from a single point as it does when there is a true focus of expansion), it may be sufficiently radial to influence neural mechanisms that are sensitive to expanding optic flow. In a previous study, we referred to the point of intersection of the trailing edge and the horizon as the *pseudo FoE* and found that human heading judgments are biased toward the pseudo FoE when it is present [24].

For the present purposes, the important feature of the pseudo FoE is that it drifts with the trailing edge of the object and is therefore not stable in the visual field (except when the trailing edge remains at a fixed direction). Nonetheless, stimuli that simulate this scenario do not induce an impression that heading is changing as if moving along a curved path. Layton & Fajen showed that the mean heading error in conditions with the pseudo FoE never exceeded 3° [24], suggesting that human heading perception is more stable and accurate than would be the case if the visual system was simply tracking the pseudo FoE.

This feature of the pseudo FoE poses a potential challenge for models of heading perception that lack temporal dynamics because such models may yield heading estimates that continually drift with the pseudo FoE as it sweeps across the visual field. Indeed, the heading estimates generated by the differential motion and motion pooling models are substantially biased toward the pseudo FoE and rapidly drift as the position of the pseudo FoE changes (Fig 6). In contrast, the heading estimate from the competitive dynamics model is far more accurate and stable.

**Fig 7:**
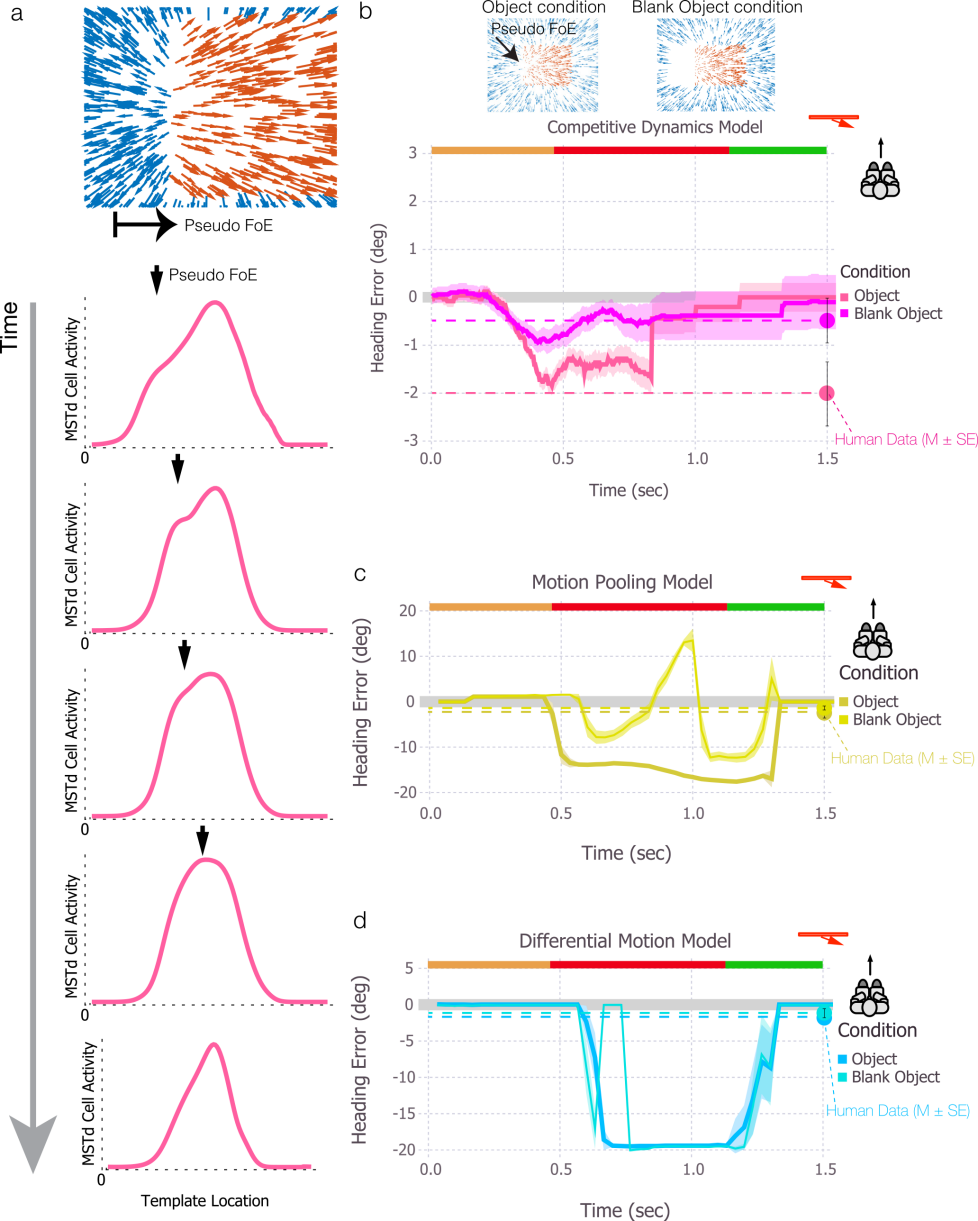
Simulations of an approaching object that creates a radial-like flow pattern nearby the object’s trailing edge (pseudo FoE). (a) Plots of MSTd unit activity in the competitive dynamics model over time. The x-axis corresponds to the preferred FoE position among the MSTd units tuned to radial expansion and the y-axis shows the activity level of each of these units. The downward arrow indicates the position of the pseudo-FoE at each point in time. The pseudo FoE creates a secondary peak to the left of heading but is largely suppressed by competitive interactions and only influences the heading estimate when it merges with the dominant peak. (b-d) Simulation results for the competitive dynamics (b), motion pooling (c), and differential motion (d) models comparing conditions with a visible pseudo FoE (Object condition) and a pseudo FoE that is partially occluded by a blank object (Blank Object condition). Error bars for the mean human heading judgments indicate ±1 SEM.

To build the reader’s intuition about how competitive dynamics within MSTd moderates the heading error in this scenario, let us examine how activity in MSTd evolves over time. Each panel below the flow diagram in Fig 7A plots the activity of MSTd units tuned to FoE positions along a one-dimensional horizontal cross-section of the visual display. Early in the trial when the object is farther way (top panel), there is a moderately broad distribution of activity with a peak that coincides with the observer’s actual heading. The asymmetry in the distribution arises due to the influence of the pseudo FoE, which produces an inflection point to the left of the heading direction. As the object nears the observer’s future path, the inflection point corresponding to the pseudo FoE response drifts closer to the centrally positioned heading peak (second panel). The height of the secondary peak increases over time, indicating an increased influence of the pseudo FoE throughout the network as the motion contrast at the trailing edge of the object becomes more radial (third panel). However, competitive dynamics within MSTd prevent the secondary peak from dominating, even when the background FoE is occluded by the object and the pseudo FoE is the most radial flow pattern in the visual field. When the pseudo FoE becomes close enough to the heading direction, the response peaks merge, yielding a new peak that is slightly shifted in the direction of the pseudo FoE (leftward; third and fourth panels). As the object moves to the side to reveal the background FoE, the MSTd activity peak drifts back to coincide with the observer’s heading (fifth panel). In summary, the model moderates heading error as the pseudo FoE sweeps across the visual field through competitive dynamics that suppress the secondary activity peak in model MSTd until the position of the pseudo FoE becomes close enough to the heading direction to influence the peak of the overall distribution.

As a direct test of the hypothesis that heading perception can be influenced by the pseudo FoE, the study by Layton & Fajen [24] also included a condition in which a blank object was placed near the trailing edge of the moving object, as depicted in the flow diagram in Fig 7B. Although the blank object occludes optic flow from the background, and background optic flow provides information about the true heading direction, heading perception should actually become more accurate since the blank object greatly diminishes the pseudo FoE. Indeed, the bias in human heading judgments that arose when the pseudo FoE was present was nearly erased when the blank object was included [24].

We simulated all three models with and without a blank object under conditions that give rise to a pseudo FoE at the trailing edge that remains stationary in the visual field. With the competitive dynamics model (Fig 7B), the weak (< 2°) heading bias caused by the pseudo FoE was roughly halved when the blank object was added, consistent with human performance. Simulations of the motion pooling model without the blank object again yielded a large (~15°) bias that coincided with the pseudo FoE (Fig 7C). When the blank object was added, the heading estimate exhibited wild fluctuations. The large positive deflection shortly before 1 sec indicates that when the pseudo FoE is eliminated, the model template response drifts with the motion contrast at the leading edge of the object, which is the next most radial-like pattern in the optic flow field. Unlike humans, the differential motion model did not show a difference in heading estimates across the Pseudo FoE and Blank Object conditions (Fig 7D).

### Optic flow from video

Our second additional test of robustness and stability uses video rather than analytic optic flow as the input signal. Flow detected from the video is inherently noisier and sparser than that that is analytically specified. Since decreases in the optic flow density do not weaken the influence of moving objects on heading judgments, this serves as an important test of model robustness.

**Fig 8:**
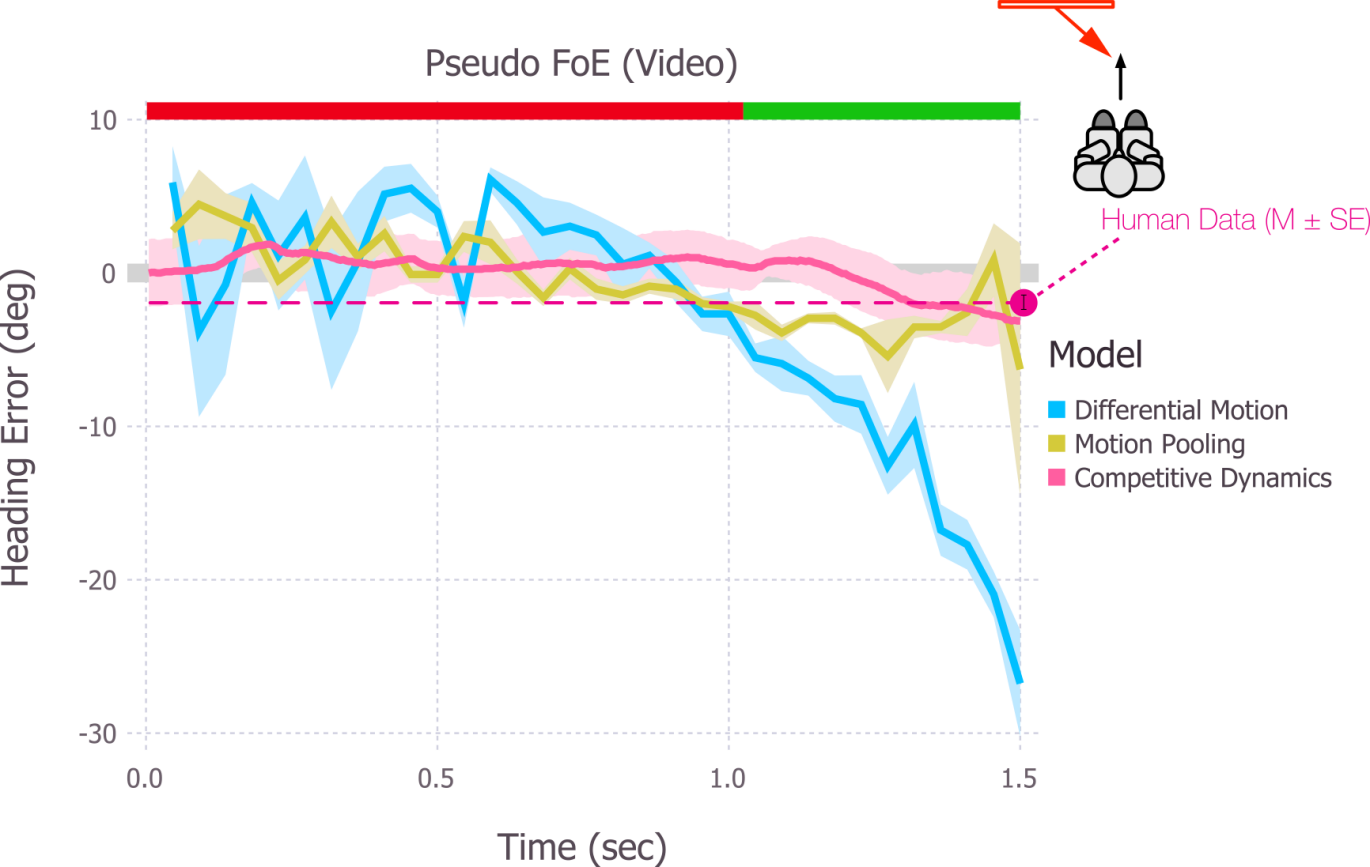
Heading estimates produced by the motion pooling, differential motion, and competitive dynamics models when a video of a pseudo FoE condition presented to the subjects in [24] served as input. Error bars for the mean human heading judgments indicate ±1 SEM.

For this set of simulations, the object approached from a 35° angle and the motion gave rise to a pseudo FoE when the object occluded the future path at the end of the trial. This differs from the stimuli used in the previous pseudo FoE simulations wherein the object crossed the future path earlier in the trial. The object was cylindrical and traveled along a ground plane, which resembled the condition from Experiment 3 of Layton & Fajen [24] when the object was laterally shifted by 0.75 m. The estimates from the differential motion model fluctuate about zero before the moving object crosses the observer’s path, and then turn sharply negative (Fig 8). The motion pooling model yields smaller biases, but also shows fluctuations throughout the trial. In contrast, mean heading error for the competitive dynamics model is close to zero until the object crosses the path, at which point a small negative error (as in human performance) emerges. These results highlight the robustness of recurrent competition that unfolds over time. Even though the density and position of the dot motion may vary considerably frame-to-frame in the optic flow detected from video (yielding changing global motion patterns), on-center/off-surround competition in the competitive dynamics model stabilizes the heading estimates.

### Recovery from brief interruptions: Full-field moving objects

**Fig 9:**
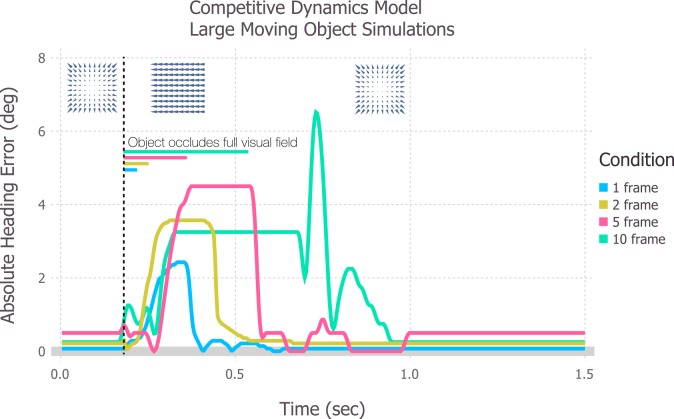
Simulations of the competitive dynamics model of self-motion through a static environment on individual trials when the entire field is replaced by 1, 2, 5, or 10 frames of laminar optic flow. The simulations reveal how large variable-length perturbations affect model performance and recovery. Colored bars indicate the duration of the optic flow disruption and the colored curves indicate the absolute heading error in single trial simulations of model MSTd.

Human heading perception is remarkably stable even when vision is temporarily interrupted, such as during eye blinks, or in the more extreme scenarios when the entire optic flow field suddenly changes, such as when large moving objects occupy most of the visual field (e.g. a train crossing a driver’s future path). To test how extreme perturbations to the optic flow field affect model heading estimates, we replaced optic flow of simulated self-motion through a static environment with full-field laminar flow for 1, 2, 5, or 10 contiguous frames (Fig 9). We did not plot the results for the motion pooling model because heading estimates were accurate, except for during the period of laminar flow when the maximal activation shifted instantaneously from the central to most laminar template. We could not simulate the differential motion model, because the laminar flow did not contain depth variation.

Fig 9 shows how full-field laminar flow perturbations affect the absolute heading error produced by the competitive dynamics model. For this set of simulations, we show individual trials (one per condition) rather than averages across multiple trials. The duration of the laminar flow perturbation had a graded effect on the maximal absolute heading error–in general, longer laminar flow perturbations yielded larger heading errors. Heading estimates restabilized in each case, but the model required longer periods of time to recover from the longer perturbations. This occurred because the recurrent mechanisms in the competitive dynamics model not only integrate the presently available optic flow, but also the response to the optic flow time history. This leads to the prediction that misperceptions in heading that may result from prolonged extreme disruptions to the optic flow field endure for a longer period of time than those caused by shorter disruptions. In general, however, the mechanisms in competitive dynamics model tolerated even the most extreme optic flow disruptions, greatly mitigating heading errors as compared to the motion pooling model.

### Competitive dynamics and locomotion through static environments

The simulations presented thus far have focused on self-motion in the presence of moving objects. However, the stability of heading also improves over time during self-motion through static environments. This was demonstrated by Layton & Fajen [23], who showed that the variability in human heading judgments was affected by the duration of the stimulus, with the greatest variability occurring when trial duration was short (150 ms) and variability decreasing and eventually reaching a plateau at 500 ms.

**Fig 10:**
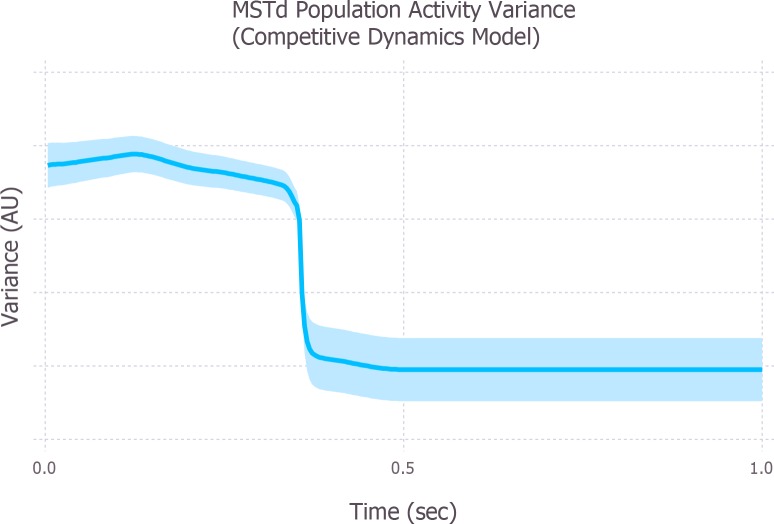
Population variance in model MSTd of the competitive dynamics model when simulating self-motion through a static environment. The variance of the MSTd population was averaged across the template space over different runs of the model. Greater variance implicates uncertainty about heading due to the broadness of the activity distribution, and less variance implicates confidence in the heading estimate due to the peaked activity distribution.

Fig 10 depicts the variance of the model MSTd population across the template-space, which indicates the quality of the heading estimate, over 1 sec of simulated self-motion through a static environment. Similar to the variability in human heading judgments, the population variance is high early in the trial, indicating uncertainty in the heading estimate due to the broadness of the MSTd activity distribution. Also like human judgments, model variance decreases over time and plateaus at approximately 500 msec. These simulations suggest that even in static environments, recurrent mechanisms within MSTd, such as those in the competitive dynamics model, may play an important role in refining and stabilizing heading estimates over time.

## Discussion

While much research has focused on the accuracy of human heading perception, the factors that underlie its hallmark robustness have been largely ignored. Heading perception is not only accurate, but remarkably robust—a property that is illuminated by research on self-motion in the presence of independently moving objects. In the present article, we focused on the question of why heading perception isn’t biased by more than a few degrees and doesn’t abruptly shift when an object crosses the observer’s future path.

Simulations of self-motion in the presence of laterally moving objects that approached, maintained a fixed-depth from, or retreated from the observer revealed that differential motion and motion pooling models yield unstable heading estimates over time, often changing abruptly when the object approached the future path. This was not surprising, considering that the model computations process optic flow in independent instants over time. Introducing temporal smoothing in the models was not sufficient to eliminate the abrupt fluctuations. On the other hand, the competitive dynamics model presented here, which contains competitive interactions among neural units in MSTd as well as temporal dynamics, exhibited gradual changes in heading estimates over the course of several hundred milliseconds after an object occluded or disoccluded the observer’s future path. The competitive dynamics model not only captures the pattern of human heading bias observed when approaching, fixed-depth, and retreating objects near the observer’s future path, but also the temporal stability and resilience that makes human heading perception robust. Recurrent competitive interactions that unfold over time among neurons in MSTd may hold the key to the stability and robustness of human heading perception.

### Role of competitive mechanisms in robust heading perception

The primary aim of this study was not merely to introduce a new model that generates more accurate heading estimates, but rather to examine the general principles that underlie robust and stable heading perception. The most obvious difference between the competitive dynamics model and the motion pooling and differential motion models is the introduction of temporal dynamics. However, this feature of the competitive dynamics model does not merely smooth the heading estimate over time. As demonstrated above, simply adding temporal smoothing to the motion pooling and differential motion models barely reduces the fluctuations in heading estimates. Rather, the competitive dynamics model integrates a collection of neural mechanisms (i.e., divisive normalization, on-center/off-surround interactions, recurrent connectivity, and thresholding) that are fundamental, ubiquitous operations performed by populations of neurons all throughout cortex [47–50]. In this section, we explain how these mechanisms offer a more principled account of the robustness and stability of human heading perception.

The MSTd network in the competitive dynamics model implements a recurrent competitive field, wherein neural interactions lead to the divisive normalization of the population activity [51]. Divisive normalization represents an important property whereby the total energy in the network is conserved and the dynamic range automatically adjusts to process the input pattern, irrespective of gain fluctuations [52]. Automatic gain control plays a particularly important role in stabilizing network dynamics in a number of scenarios, such as in the pseudo FoE video simulation, wherein the reliability of the optic flow signal may vary considerably over time.

In model MSTd, units compete via on-center/off-surround interactions for their preferred heading direction, determined by the FoE position of their receptive field template. Each unit enhances its own heading signal through recurrent self-excitation and inhibits competing heading signals through recurrent inhibition (Eq 21). Crucially, the estimated heading arises through divisive or shunting interactions that unfold over time to balance the MSTd population activity and the continuously evolving bottom-up optic flow signals. These recurrent interactions result in soft winner-take-all dynamics within model MSTd and the simultaneous representation of multiple possible heading estimates at any point in time. In both the 70° approaching object (Fig 4B) and pseudo FoE simulations (Fig 7A), soft winner-take-all dynamics led to candidate heading estimates in multiple locations. This allowed the competitive dynamics model to balance, rather than abruptly switch between, estimates based on their salience over time. A similar mechanism allowed Layton & Browning [53] to capture the effects of spatial attention on the tuning of MSTd neurons.

While others have proposed models that depend on recurrent mechanisms in MSTd [54–56], there are important differences between these models and the competitive dynamics model. First, activity normalization and feedforward/feedback signal integration occur within the competitive dynamics model as a single process, tightly linked to the network dynamics. By contrast, the other models decompose these operations into three separate stages within each model area (e.g. MSTd). Second, the continuous integration of optic flow over time represents a distinctive property only captured by the competitive dynamics model. Computations performed by other models of MSTd with recurrent mechanisms occur at equilibrium, when units reach a steady state at each point in time. The competitive dynamics model is a dynamical system that is agnostic as to whether a steady state is ever reached: units respond to both the changes in the optic flow input and the evolving state of interacting V1, MT^+^ and MSTd networks. This exclusive property of the competitive dynamics model is consistent with how MT relies on a temporal solution to overcome the aperture problem [57] and how human heading perception depends on the temporal evolution of the optic flow field [23]. The fact that optic flow acceleration/deceleration played an important role in accounting for human judgments in the analytical model of Raudies & Neumann [45] strengthens the evidence that the temporal evolution of the optic flow field plays an important role in heading perception.

Returning to the question of why heading perception does not abruptly shift when an object crosses the observer’s future path, the explanation provided by the competitive dynamics model is that the competitive dynamics among MSTd neurons take time to unfold. Optic flow signals arriving at the present time interact with the present state of the network that reflects the information about the heading direction detected over the course of the recent time history. For example, when many MSTd units are simultaneously active, there is greater uncertainty about the heading direction across the population and a reliable optic flow signal may quickly influence the heading estimate (Fig 10). On the other hand, if few MSTd units are active, meaning there is a high degree of confidence in the heading estimate across the population, it may take some time for even a strong distinct optic flow signal to influence the heading estimate. This property contrasts with the behavior of other models whereby the activation of model MSTd always reflects an instantaneous transformation of the optic flow field. The time course of competitive dynamics likely interacts with the relatively slow response latencies (median: ~190 msec) of MSTd neurons to radial expansion [58,59].

The fact that the competitive dynamics model captures human heading perception so well highlights the importance of these competitive mechanisms. The motion pooling and differential motion models implement algorithms that are sufficiently generic that they could in principle be carried out on vector field representations of the optic flow field without regard to neural systems. For example, the motion differencing operations performed by the differential motion model could just as well be carried out on raw optic flow vectors without the interpretation that MT units perform the operation. On the other hand, the competitive dynamics model explicitly models individual neurons and their interactions in dynamically evolving networks—the computations and neural interpretation are inextricably linked. We emphasize that the upshot is not that the competitive dynamics model is superior to the other models, but that the competitive mechanisms that implement more realistic neural dynamics play a central role in the robustness and stability of heading perception.

### Role of segmentation in robust heading perception

To account for human heading judgments in the presence of moving objects, the differential motion model segments the optic flow field on the basis of local speed differences [25] and the model of Raudies & Neumann [45] segments on the basis of accretion/deletion, expansion/contraction, and acceleration/deceleration. This raises the question of whether the visual system requires segmentation to perceive heading in the presence of moving objects. The fact that the competitive dynamics model captures patterns of known human heading judgments without any explicit segmentation of the optic flow field, shows that, at least in principle, segmentation may not be necessary and recurrent mechanisms in MSTd are sufficient. While segmentation likely plays a fundamental role in object motion perception [60], several lines of evidence do not support a role in heading perception. First, neurons in MSTd do not appear to extract the translational component when the optic flow field contains both translation and rotation [43], which is a core prediction of differential motion models [25,39,40,44]. Second, the MT cells that possess antagonistic surrounds that are proposed by Royden to perform the differential motion computations and that could be used to extract the accretion/deletion segmentation cue used by Raudies & Neumann do not appear to project to heading sensitive cells in MSTd [61–64]. Third, sensitivity to several of the “segmentation cues” of Raudies & Neumann may be achieved without explicit segmentation stages. Radial and spiral templates that realize the properties of MSTd receptive fields extract information from optic flow about expansion/contraction and spatial curvature, respectively. In addition, the competitive dynamics model extracts information about acceleration/deceleration and temporal curvature by integrating the optic flow field over time.

### Role of multi-sensory signals in robust heading perception

Although our focus has been on visual mechanisms that underlie robust heading perception, self-motion perception is inherently multi-sensory. The majority of heading sensitive cells in primate MSTd are primarily driven by visual input but modulated by vestibular signals [65]. That is, the heading tuning curve of MSTd neurons tends to be sharper and more selective when self-motion occurs in the presence of optic flow compared to in darkness [66]. The multi-sensory tuning of MSTd neurons may contribute to the robustness of heading perception during ordinary locomotion in a manner that goes beyond the visual mechanisms explored in existing models. For example, as many as half of MSTd neurons demonstrate an enhanced response when the visual and vestibular signals are consistent with one another (“congruent cells”) and others demonstrate a diminished response when the multi-sensory signals are in conflict (“opposite cells”) [67]. The multimodal response differences between these populations of MSTd neurons may increase the robustness of heading perception by discounting optic flow, such as that produced by a moving object, that does not agree with non-visual self-motion directions [68]. Despite the coarseness of vestibular tuning in MSTd, it may be sufficient to resolve whether a region of optic flow within the visual field arises due to self- or object motion [10,69] and allow heading perception to persist when optic flow is intermittently unavailable or unreliable. The availability of proprioception during active self-motion likely provides another redundant signal to facilitate heading perception [4]. More neurophysiological and modeling work needs to be performed to clarify how multisensory signals interact to contribute to the robustness of heading perception.

### Conclusions

In the present article, we simulated biological models of heading perception to investigate why perceived heading in humans is only biased by several degrees in the presence of moving objects and why the perceived heading does not abruptly shift when the object crosses the observer’s future path. We found that passive temporal smoothing alone was not sufficient in accounting for the characteristic robustness of human heading perception. However, recurrent competitive interactions that unfold over time among model units in area MSTd resulted in stable heading estimates.

## Methods

### Visual displays

We generated 1.5 sec (45 frame) sequences of optic flow that simulated self-motion toward two frontoparallel planes initially positioned 800 cm and 1000 cm away in depth from the observer. This environment was selected to accommodate the differential motion model, which performs best when the scene contains depth discontinuities. Each plane consisted of 3000 dots and occupied the entire visual field at the outset. The observer moved along a straight-ahead heading at 200 cm/sec.

In sequences that contained a moving object, the object was rectangular (150 cm x 150 cm) and consisted of 320 dots. Its trajectory was parameterized in terms of a starting lateral offset from the observer’s path, starting relative depth to the observer, speed, and heading-relative trajectory angle. Table 1 specifies the parameters of object trajectories.

**Table 1:** Parameters that define object initial position and motion relative to the starting position of the observer.

Object condition	Lateral offset (cm)	Depth (cm)	Speed (cm/sec)	Trajectory Angle
Approach 15° (Figs 2A and 4A)	100	900	200	15°
Approach 70° (Figs 2B, 3 and 4B)	400	600	200	70°
Fixed-Depth (Figs 2C and 4C)	200	250	200	-45°
Retreating (Figs 2D and 4D)	150	100	300	-56°
Pseudo FoE (Fig 6)	150	400	200	70°
Pseudo FoE (Fig 7)	170	600	200	45°

In each scenario, the object started on the left hand side of the observer and moved rightward. Approach and retreat from the observer is indicated by positive and negative angles, respectively.

In the video simulation, we detected optic flow in the video (320 px x 240 px) using the Horn-Schunk algorithm built into MATLAB’s computer vision toolbox, which served as the input to the differential motion and motion pooling models [70]. The competitive dynamics model detected motion from the video directly. Note that the Horn-Schunk optimization includes a regularization term in the objective function that smoothes the estimated motion field. Compared to correlational [71] or motion energy [72] approaches to motion detection, which do not require smoothing of the motion vector field, the Horn-Schunk algorithm should generate motion and heading estimates in motion pooling and differential motion models that are no less stable and accurate.

To ensure that the variability is not due to an insufficient sample size, we ran each of the models on a number (usually 25) of the optic flow sequences that were identical except for the initial random placement of the dots until the variance in the heading estimates plateaued.

We also simulated all three models in a static environment (no moving object) to confirm that they were operating as expected and that there were no systematic biases. These simulations revealed a mean heading error close to zero with low variability for all three models.

### Differential motion and motion pooling models

The differential motion [25] and motion pooling [20] models were implemented in MATLAB according to their published specifications. We changed several parameters to ensure that the models performed as expected on our visual displays. For example, we set the model MSTd Gaussian pooling variance parameter (σ) to 19 px in the model of Royden (2002) to ensure that heading estimates were in the direction of object motion for the fixed-depth object and in the direction opposite object motion for the approaching object. We changed the same parameter in the model of Warren & Saunders [20] to 25 px to achieve the best performance across the visual displays that we tested. Model MT in both models consisted of units that tiled the input dimensions of the visual displays. To generate the opponent operators with different differencing axes in the model MT of Royden [25], we filtered the input with 7x7 rectified Gabor kernels. MSTd units had overlapping receptive fields and were centered every two pixels along each of the input dimensions. Parameter values remained the same in all models and simulations. Analytical optic flow computed by a pinhole camera projection served as input to the differential motion and motion pooling models [38]. We derived model heading estimates by considering the location of preferred FoE of the maximally active MSTd unit along the horizontal cross-section that contained the observer’s heading. Heading error was computed by subtracting the location of the preferred FoE of the maximally active MSTd unit from that which coincides with the observer’s heading direction.

### The competitive dynamics model

The model presented here encompasses the V1-MT^+^-MSTd processing stages of the Layton et al. model [28]. Updates have been performed to the front-end so that the model detects optic flow from video input using stages that correspond to those along the primate magnocellular pathway. Moreover, algorithmic simplifications used in the Layton et al. model have been replaced so that each stage consists of networks of coupled Hodgkin Huxley type ordinary dynamical equations. Our model builds on the STARS and ViSTARS models [73–75].

**Fig 11:**
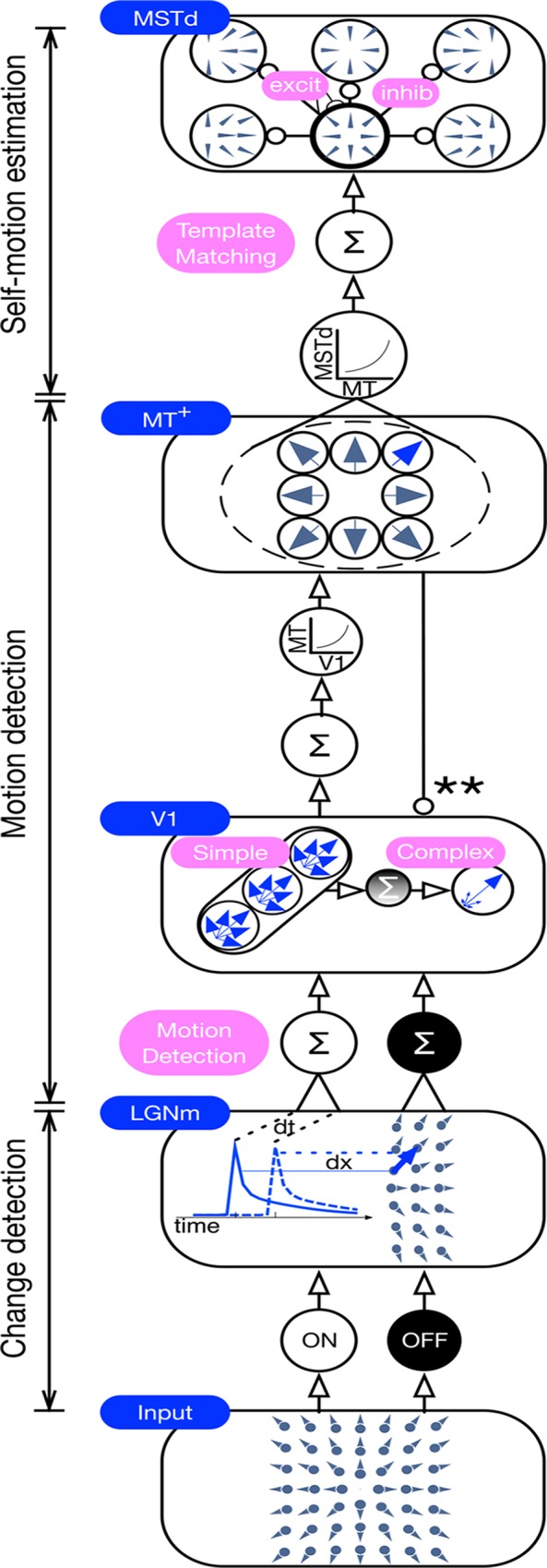
Overview of the competitive dynamics model. The model consists of three main stages: change sensitivity (model Retina and LGN), motion detection (model V1 and MT^+^), and self-motion estimation (model MSTd). Units in model LGN respond to frame-to-frame pixel changes in the video input. The model generates an initial estimate of motion speed and direction in V1 simple cells, which receive input from spatially offset LGN units with a number of conduction delays. Similar to the motion sensitivity of cells in primate, the direction tuning at the stage of model simple cells is broad and coarse. The motion estimate is refined through spatial on-center/off-surround grouping of the motion signals by complex cells. Consistent motion signals are grouped over a larger spatial scale by units in MT^+^, which send feedback to inhibit complex cells that differ in direction selectivity. The activity distribution in MT^+^ is matched against a number of radial expansion and contraction templates with varying FoE/FoC positions, which serves as the input to model MSTd. Units in MSTd tuned to radial expansion and contraction with different preferred singularity positions compete with one another to resolve the self-motion direction. Pooling stages in the model are depicted by ‘Σ’ and the curve indicates thresholding and squaring operations.

**Fig 12:**
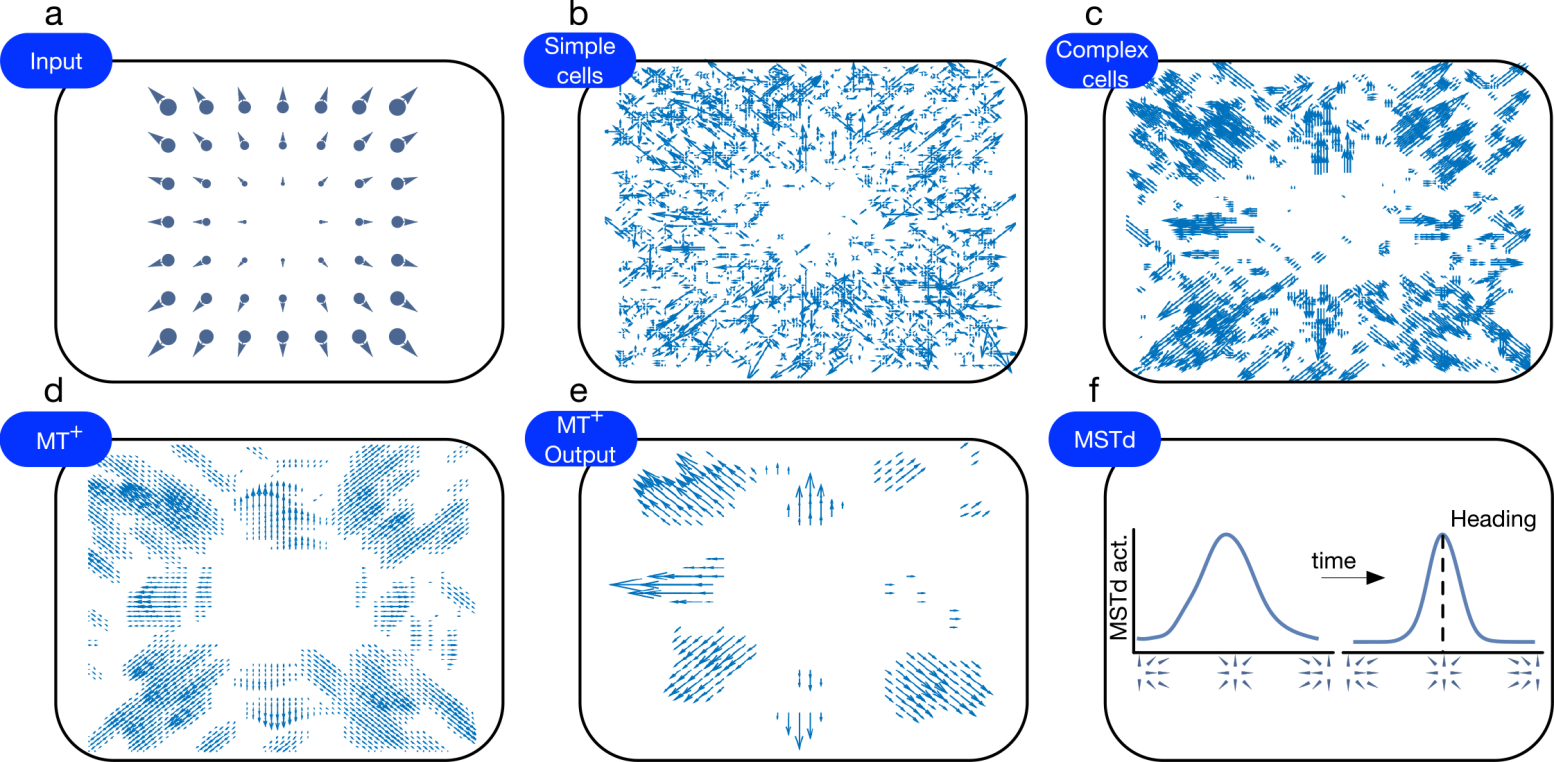
**Plots of the different stages in the competitive dynamics model when simulating self-motion through a static environment (a).** (b-e) The response of V1 and MT^+^ cells were mapped onto a vector field using population vectors. Each vector was created by summing a collection of unit vectors oriented in each unit’s preferred direction and weighted according to the firing rate of units whose receptive fields overlap. (f) The response of heading-selective MSTd cells at the beginning (left) and end (right) of the trial. Competition sharpens the population activity over time, increasing the certainty about the estimated heading direction.

Fig 11 schematically depicts an overview of the model. The model consists of three main stages: detecting changes in luminance (model Retina and LGN), detecting motion (model V1 and MT^+^), and estimating self-motion (model MSTd). The details of these stages are described in the following sections. Fig 12 shows the response of each model area to simulated self-motion in a static environment toward two frontoparallel planes.

### Mathematical notation

The ordinary differential equations described in the following sections model the dynamics of cells across multiple brain areas along the magnocellular pathway of primate cortex. Equations often assume the form of a recurrent competitive field [51]:

$$\frac{{dx}_{i}}{dt}=-x_{i}+(1-x_{i})(f(x_{i})+I_{i}^{+})-x_{i}(\sum_{k\neq i}f(x_{k})+I_{i}^{-})$$ (1)

The firing rate *x* of unit *i* in the network layer described by Eq 1 obeys shunting dynamics, which implement a number of important dynamical properties, such as divisive normalization and boundedness [51,52,76]. Eq 1 contains a passive decay term −*x*_*i*_, excitatory input term $\left(f(x_{i})+I_{i}^{+}\right)$ that is shunted by (1 − *x*_*i*_) to ensure the firing rate remains bounded above by 1, and inhibitory input term $\left(\sum_{k\neq i}f(x_{i})+I_{i}^{-}\right)$ that is shunted by *x*_*i*_ to bound the firing rate from below by 0. The variables $I_{i}^{+}$ and $I_{i}^{-}$ denote excitatory and inhibitory inputs to unit *i*, respectively. In Eq 1, the function *f*(*x*_*i*_) regulates the recurrent self-excitation or inhibition that the unit receives from others in the same network. In model networks, *f* is a sigmoid function that gives rise of soft winner-take-all dynamics [51,77]. We use the sigmoid

$$f(w;f_{0})=\frac{w^{2}}{w^{2}+f_{0}^{2}}$$ (2)

that achieves its half-maximum value of 0.5 at *f*_0_.

The output of network layers may be thresholded by *Γ* according to the following function *g*

$$g(w;\Gamma )={\left[w-\Gamma \right]}^{+}$$ (3)

where the notation [∙]^+^ indicates the half-wave rectification max(∙,0).

Network equations apply to all units in the layer and as such we use matrix bold notation. For example, ***x*** denotes the array of cells at each spatial location (*i*,*j*). Connectivity between units in different network layers are either connected 1-to-1 or through a Gaussian kernel that defines the convergence of feedforward or feedback signals. When units are connected 1-to-1, the unit with a receptive field centered at position (*i*,*j*) projects to a unit in another layer at position (*i*,*j*). The Gaussian kernel ***G***_*σ*,*s*_ defines how connections converge from one layer onto the next when the spatial extent of the receptive field is larger than that of its input.

Gσ,s=exp(−x∙x2σ2)∑i∑jexp(−x∙x2σ2) (4)

In Eq 4, the operator ∙ defines the dot product, *σ* indicates the standard deviation, and *s* defines the radius of the kernel. Convolutions in the following sections are always centered at the position of each unit (*i*,*j*). In some cases, the Gaussian kernel that has radius *s* and is elongated in the direction *d* corresponding to the angle *θ*, which we define as $W_{\bar{\sigma },s,d}$.

Wσ¯,s,d,i,j=exp(−∇(i−xj−y)TΣ−1∇(i−xj−y)2) (5)

In Eq 5, **∇** is the rotation matrix $\nabla =\left[\begin{array}{cc} \cos\theta & \sin\theta \\ -\sin\theta & \cos\theta \end{array}\right]$, θ=d4, $\Sigma =\left[\begin{array}{cc} \sigma_{x} & 0\\ 0 & \sigma_{y} \end{array}\right]$, and $\bar{\sigma }=\sigma_{x}/\sigma_{y}$. The kernel $W_{\bar{\sigma },s,d}$ is normalized to sum to unity.

Units in areas LGN and V1 uniformly tiled 1-to-1 the spatial dimensions of the visual display. The overlapping receptive fields of MT^+^ and MSTd units were spaced according to their speed sensitivity. Units in MT^+^ tuned to speeds of 1, 2, and 3 px/frame had receptive fields uniformly distributed throughout the visual input array at every single, second, and third pixel, respectively. MSTd units had receptive fields centered every 6 px, doubling the maximum offset of MT^+^.

### Model retina

The pattern of luminance at time *t* in the visual signal ***I***(*t*) is transformed into signals of increments ***J***^+^(*t*) and decrements ***J***^−^(*t*), which represent the change in the input across successive frames. These signals correspond to the coding of luminance increases and decrease by ON and OFF retinal ganglion cells.

$$J^{+}(t)=[{I(\left\lceil t\right\rceil )-I(\lceil t-1\rceil )]}^{+}$$ (6)

$$J^{-}(t)=[{I(\left\lceil t-1\right\rceil )-I(\lceil t\rceil )]}^{+}$$ (7)

The notation ⌈∙⌉ refers to taking the ceiling of the operand.

### Model LGN

Model LGN units ***L***^±^(*t*) respond to transient changes in the visual signal, but are not direction selective [78]. ON and OFF LGN units remain sensitive to the luminance increments and decrements in their retinal inputs, respectively [79]. The following equation describes the activity of LGN units:

$$L^{\pm }(t)=[{R^{\pm }(t)Z^{\pm }(t)]}^{+}$$ (8)

where ***R***^±^ indicates the population of units that perform a leaky integration of its retinal inputs, which is gated by the habituative transmitter ***Z***^±^. Habituative gates, sometimes called dynamic or depressing synapses, prevent responses to persistent inputs by modeling the slow-term deletion of a neuron’s neurotransmitter stores [80,81]. Tonic inputs depress the habituative gates, which when multiplicatively combined with ***R***^±^, lead to rapid suppression of the LGN activity ***L***^±^. Habituative gates slowly recover to their full capacity in the absence of input. In effect, ***L***^±^ responds well to motion and weakly to stationary inputs.

$$\frac{dR^{\pm }}{dt}=\epsilon_{LGN,R}(-R^{\pm }+(1-R^{\pm })J^{\pm })$$ (9)

$$\frac{dZ^{\pm }}{dt}=\epsilon_{LGN,Z}(1-R^{\pm }-\lambda (R^{\pm }Z^{\pm }))$$ (10)

In Eqs 9 and 10, *ϵ*_*LGN*,*R*_ corresponds to the inverse time constant of each cell ***R***^±^, *ϵ*_*LGN*,*Z*_ corresponds to the inverse time constant of each gate ***Z***^±^, and *λ* indicates the transmitter depletion/repletion rate. For our simulations, we fixed *ϵ*_*LGN*,*R*_ = 2 *sec*^−1^, *ϵ*_*LGN*,*Z*_ = 0.01 *sec*^−1^, and *λ* = 10.

### Motion detection

**Fig 13:**
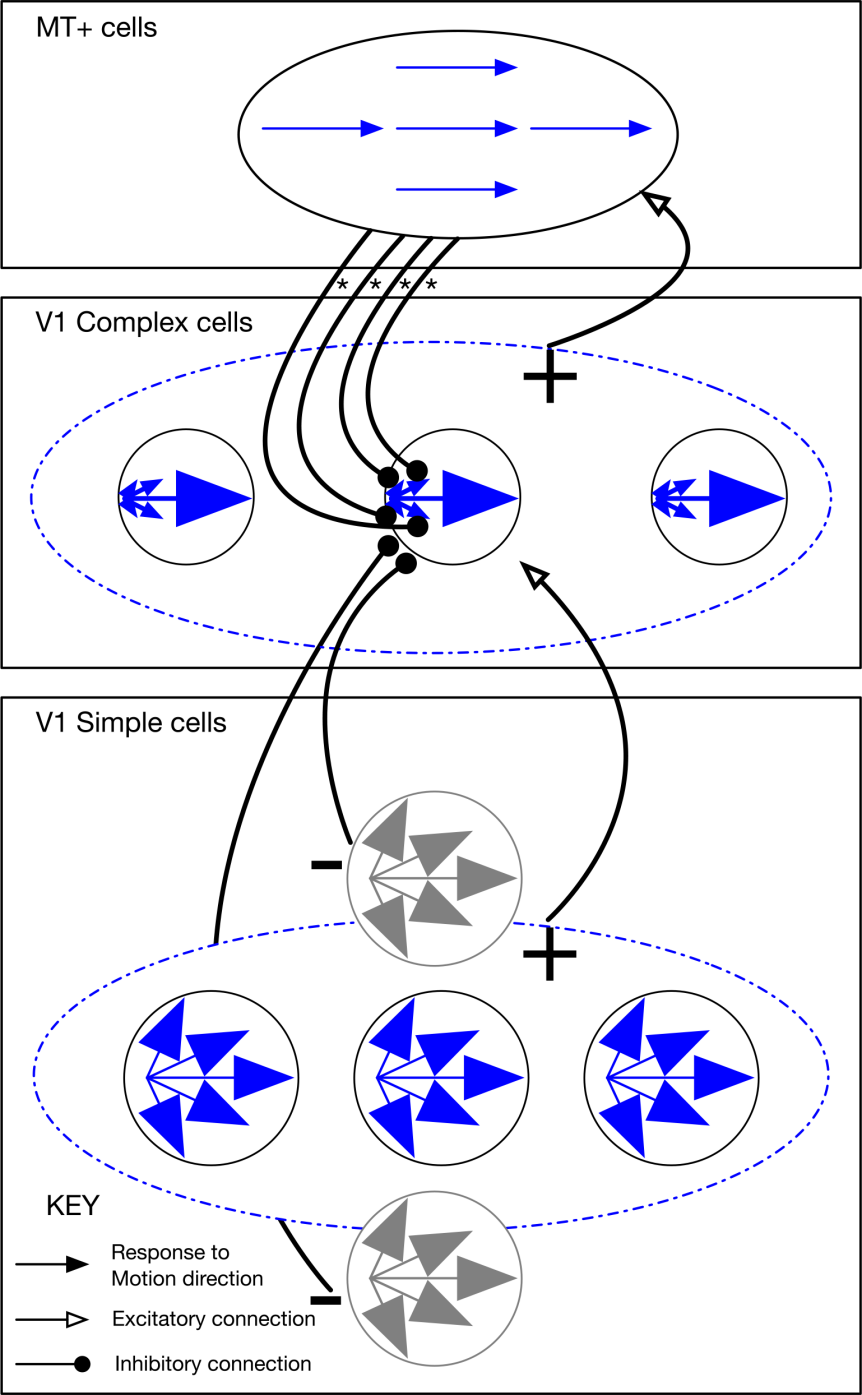
Diagram of the model V1–MT^+^ motion detection microcircuit. V1 complex cells receive on-center/off-surround input from simple cells, broadly tuned to motion. Simple cells are grouped by complex cells parallel to their preferred direction of motion, and simple cells positioned perpendicular to the preferred direction inhibit the complex cell. MT^+^ units refine the motion estimates by grouping over a larger spatial extent, and sending feedback to inhibit complex cells with discrepant direction selectivities in the receptive field.

The detection of motion direction occurs through a three stage process that corresponds to simple and complex cells in V1, and cells in area MT^+^ with excitatory surrounds (Fig 13). First, motion is detected by simple cells using a Reichardt or correlation-based mechanism based on the arrival of signals from LGN with different conduction delays and receptive field locations [71] (but see [73,74,82–84] for an alternative biological mechanism that relies on nulling inhibition). The motion signal is refined through short-range feedforward on-center/off-surround pooling of simple cell activity by complex cells (Fig 13, bottom two panels). Finally, a feedback loop between V1 complex cells and MT^+^ cells disambiguates local motion signals (i.e. solves the aperture problem) through the spatial pooling of complex cells by units in MT^+^ tuned to the same motion direction and the suppression of complex cells tuned to dissimilar motion directions (Fig 13, top two panels).

An asterisk appears adjacent to the inhibitory feedback connections from MT to V1 in Fig 13 because the model proposes that the net effect on V1 complex cells is inhibitory, not that the individual feedback projections are inhibitory. In fact, feedback projections to V1 are likely mostly excitatory and target excitatory neurons [85]. MT feedback projections target several V1 layers, including layer 6 [86], and neurons therein project to inhibitory interneurons [87] and those involved in feedforward processing [88] in layer 4C. Given that layer 4 neurons project to layer 2/3 [89], which contains complex cells, the circuit may serve a modularity role on complex cells. Even though the MT-V1 feedback projections may be excitatory, they may exert an inhibitory effect on complex cells, consistent with predictions from the competitive dynamics model. Modeling the laminar microcircuitry of V1 extends beyond the aims of the present paper, so we use inhibitory MT feedback as a simplification (see [90] for a laminar model of V1).

The feedback loop between MT and V1 proposed by Bayerl & Neumann [91] bears some similarity to the one described here, but, contrary to the predictions of the competitive dynamics model, the feedback exerts an excitatory rather than an inhibitory influence on V1 units. Feedback signals from MT that suppress complex cells sensitive to dissimilar motion directions rather than enhance complex cells sensitive to similar motion directions prevent the biologically implausible scenario of a runaway positive inter-areal feedback loop between V1 and MT and are consistent with the reduced suppression in V1 neurons following MT inactivation [92,93].

In the following sections, we describe the details of model areas V1, MT and MSTd.

### Model V1 (simple cells)

Signals from model LGN with spatially displaced receptive field centers ($\vec{\Delta }$) that possess a spectrum of conduction delays ($\vec{\delta }$) converge onto V1 [94]. In other words, each V1 unit acquires its speed and direction tuning based on the spatiotemporal correlation present in its convergent afferent LGN signals. For simplicity, we consider the two conduction delays *δ*_0_ and *δ*_1_ that implicate motion detection across successive frames: *δ*_0_ = ⌊*t*⌋ and *δ*_1_ = ⌊*t* − 1⌋, where ⌊∙⌋ indicates taking the floor of the operand.

V1 units were tuned to speeds (*s*) of 1, 2, or 3 px/frame in the 8 cardinal directions (*d*): up, down, left, right, and the four diagonals. The speed-direction tuning was determined by summing the LGN signal centered at position (*i*,*j*) derived from the present time with the delayed signal centered at position (*i* − Δ_*x*_,*j* − Δ_*y*_) derived from the optic flow on the previous frame. We use the set Φ(s,d) to refer to the nonzero spatial offsets from position (*i*,*j*) that implicate motion with speed s in the direction d. For example, a V1 simple cell tuned to unit speed motion in the rightward direction would have an offset $\vec{\Delta }\in \Phi (1,1)$, where $\vec{\Delta }=(-1,0)$. In other words, comparing the delayed signal displaced one unit to the left to the present signal yields sensitivity to rightward motion. To account for the increased distance along the diagonals, we scaled these signals by a factor of $\sqrt{s}$. To ensure that the V1 unit receives input from the most correlated input (Eq 10), the signal is thresholded with *Γ*_*LGN*_ = 0.45 and squared [48,72]. The set of spatial offsets $\left|\vec{\Delta }\in \Phi (s,d)\right|$ grows with speed, but not all offsets factor into the output signal, so we normalized by the number of active contributions $\left(\frac{1}{\left|A_{s,d}^{\pm }>0\right|}\right)$ (Eq 11). This adaptive normalization approximates the homeostatic plasticity process known as synaptic scaling [95].

The bottom-up input ***B***^±^ to V1 is computed based on the afferent LGN signals ***L***^±^ according to the following equations:

$$A_{s,d,i,j}^{\pm }(t,\vec{\Delta })={{\left[L_{i,j}^{\pm }(\delta_{0})+L_{i-\Delta_{x},j-\Delta_{y}}^{\pm }(\delta_{1})-\Gamma_{LGN}\right]}^{+}}^{2}$$ (11)

$$B_{s,d,i,j}^{\pm }(t)=\frac{1}{\left|A_{s,d}^{\pm }>0\right|}\sum_{\vec{\Delta }\in \Phi (s,d)}A_{s,d,i,j}^{\pm }(t,\vec{\Delta })$$ (12)

Simple cells in model V1 perform a leaky integration of their adaptive spatiotemporal inputs

$$\frac{dS_{s,d}^{\pm }}{dt}=\epsilon_{S}(-S_{s,d}^{\pm }+(1-S_{s,d}^{\pm })B_{s,d}^{\pm })$$ (13)

where the units have an inverse time constant *ϵ*_*S*_ = 5 *sec*^−1^.

Fig 12B shows the pattern of activity of direction selective simple cells tuned to unit speed at the end of a self-motion simulation through a static environment.

### Model V1 (Complex Cells)

Model complex cells are tuned to a direction and speed, but unlike simple cells, are insensitive to contrast polarity. Complex cell units refine and enhance their directional selectivity by locally pooling over simple cells with the same direction and speed tuning parallel to the preferred motion direction (Fig 13). The units also receive shunting inhibition from nearby simple cells whose receptive fields are positioned in the orthogonal direction. This feedforward connectivity between model simple and complex cells enhances responses to uniformly moving dots or surfaces and implements the collinear facilitation and orthogonal suppression properties of V1 complex cells [96,97].

V1 complex cell units compete across preferred motion direction in the following contrast enhancing network:

$$Q_{s,d}={g(S_{s,d}^{+}+S_{s,d}^{-},\Gamma_{C})}^{2}$$ (14)

$$\frac{dC_{s,d}}{dt}=-C_{s,d}+\left(1-C_{s,d}\right)\left(C_{s,d}^{2}+\sum_{\left(I,J\right)}(W_{{\bar{\sigma }}_{MT},s,\theta ∥d,i-I,j-J}Q_{s,d,I,J})\right)$$

$$-C_{s,d}\left(\sum_{k\neq d}(C_{s,k}^{2}+Y_{s,k})+\sum_{\left(I,J\right)}(W_{{\bar{\sigma }}_{MT},s,\theta ⊥d,i-I,j-J}Q_{s,d,I,J})\right).$$ (15)

In Eq 15, $W_{{\bar{\sigma }}_{MT},s,d}$ is the anisotropic Gaussian kernel elongated in the direction *d* (${\bar{\sigma }}_{MT}=15$), the notation *θ* ∥ *d* means the angle *θ* of the preferred motion direction, the notation *θ* ⊥ *d* means the angle $\theta +\frac{\pi }{2}$ orthogonal to the preferred motion direction, and ***Y*** defines the feedback a complex cell receives from area MT^+^ (see Eq 18):

$$Y_{s,d}=\sum_{\left(I,J\right)}\left(G_{\sigma_{V1},3s,i-I,j-J}M_{s,d,I,J}\right)$$ (16)

where *σ*_*V*1_ = 0.5. Complex cells receive inhibitory feedback from model area MT^+^ that suppresses the activity of units with preferred directions *k* that differ from the complex cell preferred direction *d*. Note that each matrix ***M***_*s*,*d*_ has been scaled back into the coordinate system of the visual display before performing the convolution.

Fig 12C depicts the pattern of activity of complex cells tuned to unit speed at the end of the self-motion simulation.

The complex cell output from V1 ***O***_*s*,*d*_ is thresholded by *Γ*_*v*1*mt*_ = 0.01, squared, and transformed by the sigmoid function *f*(∙;*γ*_*v*1*mt*_), with an inflection point *γ*_*v*1*mt*_ = 0.01.

$$O_{s,d}=f\left({g(C_{s,d},\Gamma_{v1mt})}^{2},\gamma_{v1mt}\right)$$ (17)

### Model MT^+^

Units in model MT^+^ perform a long-range spatial pooling over complex cell signals. These cells inherit their directional tuning from V1 complex cells, but integrate motion within their larger receptive fields. Analogous to V1 complex cells, the receptive field of units in MT^+^ is elongated in the direction parallel to preferred motion direction [97].

$$\frac{dM_{s,d}}{dt}=-M_{s,d}(1-M_{s,d})\left(\sum_{\left(I,J\right)}\left(W_{{\bar{\sigma }}_{MT},s,\theta ∥d,i-I,j-J}O_{s,d,I,J}\right)\right)$$ (18)

Units in MT^+^ respond well when complex cells with a consistent directional tuning are active within the receptive field (Fig 13, top panel). Over time, the suppressive feedback loop between V1 and MT^+^ resolves ambiguity in the detected motion direction (Fig 13, top two panels). Fig 12D depicts the MT^+^ cells tuned to unit smallest scale at the end of the self-motion simulation.

The output signal ***N***_*d*_ from MT^+^ collapses across speed, after scaling each matrix ***M***_*s*,*d*_ into the coordinates of MSTd. Units are collinearly pooled, parallel to their preferred motion direction, thresholded with *Γ*_*MT*_ = 10^−3^, and subsequently squared:

$$N_{d}=g{\left(\sum_{\left(I,J\right)}\left(W_{{\bar{\sigma }}_{MT},2,\theta ∥d,i-I,j-J}\sum_{s}M_{s,d,I,J};\Gamma_{MT}\right)\right)}^{2}$$ (19)

Fig 12E shows the output signal from MT^+^.

### Model MSTd

Although a full characterization of MSTd neuron receptive fields remains an ongoing challenge [48], the set of spiral templates spanned by linear combinations of radial and circular motion patterns has proven successful in capturing the pattern sensitivity across the population [98]. Analyses performed with a prior version of the competitive dynamics model revealed that linear self-motion did not activate spiral templates; such templates may play an important role in perceiving self-motion when traveling along a curved path [77]. Even in the presence of objects moving along a linear trajectory, the optic flow does not contain the spatial or temporal curvature required to activate spiral templates [45]. Because the range of conditions considered in the present study focus on observer and object motion along linear trajectories, we restricted the model MSTd template space to radial patterns.

Our model MSTd does not include cells with sensitivity to laminar or “planar” flow [14], but the role of such cells should be investigated in future research. Whereas objects that approach the observer along nearly parallel trajectories generate radial motion patterns within the object contours (Fig 1A), fixed-depth objects (e.g. Fig 1B) and objects that approach with large path angles generate more laminar flow patterns. Through potential interactions with radial cells, activation of cells in MSTd tuned to laminar flow by these moving objects may stabilize heading signals and reduce bias. In this regard, cells tuned to laminar flow may participate in a redundant mechanism that complements or factors into the recurrent competition used in the competitive dynamics model. Future physiological and modeling work should clarify the role of cells tuned to laminar flow for heading perception, particularly in the presence of moving objects.

Model MSTd matches an array of templates ***T***^±^ tuned to radial expansion (+) and contraction (-) with the bottom-up signal from model MT^+^ ***N***_*d*_. The templates $T_{i,j}^{\pm }$ have FoE/FoC positions centered on every position (*i*,*j*) within the spatial grid tiling of MSTd. The specification of expansive and contractive templates has been described previously [77]. In the following equation, the match is normalized by the energy of each template:

$$V_{i,j}^{\pm }=\frac{1}{\sum_{\left(I,J\right)}\left(T_{i,j,I,J}^{\pm }\right)}\sum_{d}\sum_{\left(I,J\right)}\left(T_{i,j,I,J}^{\pm }N_{d,I,J}\right)$$ (20)

MSTd cells compete in a soft winner-take-all network across the polarity of radial motion (expansion versus contraction) and over 2D space: Each cell has a firing threshold *Γ*_*MST*_ = 0.3 and sigmoid inflection point *f*_*MST*_ = 0.001. The kernel $G_{\sigma_{MST},7}$ specifies how units with neighboring singularity selectivities compete with one another, with *σ*_*MST*_ = 10. The dynamics of the MSTd cell tuned to radial expansion or contraction with FoE/FoC selectivity at position (*i*,*j*) is defined as follows:

$$\frac{dP_{i,j}^{\pm }}{dt}=-P_{i,j}^{\pm }+(1-P_{i,j}^{\pm })(f(g(P_{i,j}^{\pm };\Gamma_{MST});f_{MST})+V_{i,j}^{\pm })$$

$$-P_{i,j}^{\pm }\left(\sum_{w\neq \pm }\sum_{\left(n,m)\neq (i,j\right)}G_{\sigma_{MST},7,n,m}f(g(P_{n,m}^{w};\Gamma_{MST});f_{MST})\right)$$ (21)

Fig 12F shows the activity of expansion-sensitive MSTd cells at two different points in time. The heading estimate h* from model MSTd is determined by considering the FoE selectivity of the most active cell tuned to expansion along the horizontal cross-section *x* that contained the heading direction. While other expansion-selection units were often active, the most active unit had a centrally positioned FoE selectivity due to the vertical radial symmetry of the optic flow displays simulated.

$$h^{*}=\begin{array}{c} argmax\\ x \end{array}P_{x}^{+}$$ (22)
